# Metal-based nanoparticle in cancer treatment: lessons learned and challenges

**DOI:** 10.3389/fbioe.2024.1436297

**Published:** 2024-07-11

**Authors:** Ali Hheidari, Javad Mohammadi, Maryam Ghodousi, Mohammadreza Mahmoodi, Sina Ebrahimi, Esmail Pishbin, Abbas Rahdar

**Affiliations:** ^1^ Department of Mechanical Engineering, Islamic Azad University, Science and Research Branch, Tehran, Iran; ^2^ School of Mechanical Engineering, Sharif University of Technology, Tehran, Iran; ^3^ Department of Engineering Science and Mechanics, The Pennsylvania State University, University Park, PA, United States; ^4^ Bio-microfluidics Lab, Department of Electrical Engineering and Information Technology, Iranian Research Organization for Science and Technology, Tehran, Iran; ^5^ Department of Physics, University of Zabol, Zabol, Iran

**Keywords:** cancer research, metal nanoparticle, drug delivery, hyperthermia, clinical translation criteria, metallic nanoparticles, non-metallic nanoparticles, targeting and delivery

## Abstract

Cancer, being one of the deadliest diseases, poses significant challenges despite the existence of traditional treatment approaches. This has led to a growing demand for innovative pharmaceutical agents that specifically target cancer cells for effective treatment. In recent years, the use of metal nanoparticles (NPs) as a promising alternative to conventional therapies has gained prominence in cancer research. Metal NPs exhibit unique properties that hold tremendous potential for various applications in cancer treatment. Studies have demonstrated that certain metals possess inherent or acquired anticancer capabilities through their surfaces. These properties make metal NPs an attractive focus for therapeutic development. In this review, we will investigate the applicability of several distinct classes of metal NPs for tumor targeting in cancer treatment. These classes may include gold, silver, iron oxide, and other metals with unique properties that can be exploited for therapeutic purposes. Additionally, we will provide a comprehensive summary of the risk factors associated with the therapeutic application of metal NPs. Understanding and addressing these factors will be crucial for successful clinical translation and to mitigate any potential challenges or failures in the translation of metal NP-based therapies. By exploring the therapeutic potential of metal NPs and identifying the associated risk factors, this review aims to contribute to the advancement of cancer treatment strategies. The anticipated outcome of this review is to provide valuable insights and pave the way for the advancement of effective and targeted therapies utilizing metal NPs specifically for cancer patients.

## 1 Introduction

As per annual reports, cancer was responsible for the highest number of global deaths in 2020, resulting in around 10 million fatalities (W.H. Organization, 2020; Sung et al., 2021). The emergence of nanotechnology has revolutionized various fields, including cancer research and treatment (Chaturvedi et al., 2019; Souri et al., 2022a; Najafiyan et al., 2024; Tangsiri et al., 2024). Nanotechnology has the capability to rapidly detect diverse molecular signals and biomarkers, leading to advancements in early detection, diagnostics, prognostics, and treatment strategies (Dessale et al., 2022; Laraib et al., 2022; Bakhtiari et al., 2023). The field of cancer research has made groundbreaking advancements that address common challenges associated with traditional medications, such as their imprecise distribution in the body, limited solubility in water, and restricted effectiveness (Souri et al., 2022b; Heidari et al., 2023). The utilization of nanotechnology enables highly sensitive and specific measurements, along with the ability to perform multiplexed assessments (Timilsina et al., 2022). By utilizing nanoparticle (NP)-based drug delivery systems, numerous advantages are offered over conventional methods. These systems can enhance the effectiveness of drugs and proteins by prolonging their lifespan, enhancing the solubility of drugs, and enabling precise controlled drug release at specific locations. Unlike conventional approaches, NP-based drug delivery systems provide these benefits, leading to improved therapeutic outcomes (Shamloo et al., 2021; Shamloo et al., 2022; Liu et al., 2023; Shamloo et al., 2023).

Metal NPs have gained substantial attention due to their versatile properties, making them promising candidates for various applications, in particular cancer treatment (Table 1) (Izhar et al., 2022). These include iron-/iron oxide-, copper-, gold-, cerium oxide-, silver-, calcium-, magnesium-, titanium-, barium-, nickel-, zinc-, and bismuth-based NPs, as documented in scientific literature (Khursheed et al., 2022; Xu et al., 2022). Metal NPs play a significant role in contemporary cancer research platforms, attracting increasing interest in this area. A comparative analysis of metallic NPs indicates that gold NPs (AuNPs) exhibit superior characteristics, positioning them at the forefront of research (Bansal et al., 2020). Other metal NPs, such as silver NPs (AgNPs), have also demonstrated promising performance, similar to AuNPs (Ali et al., 2023). Ongoing investigations, encompassing preliminary studies and preclinical trials, have demonstrated the promising role of metal NPs in cancer treatment (Sharma et al., 2018; Huang H. et al., 2020). The utilization of metal-based cancer therapy holds promise for advancing cost-effective treatment options, potentially surpassing the high costs associated with traditional therapies (Shi et al., 2020).

**TABLE 1:** Comparing the special advantages of metal NPs with non-metal NPs in the field of cancer treatment.

Criteria	Metallic NPs	Non-metallic NPs
Targeting and delivery	High precision in targeting tumors due to magnetic properties	EPR effect
Imaging	Superior imaging capabilities (e.g., MRI and CT)	Limited to specific imaging techniques (e.g., fluorescence)
Therapeutic properties	Intrinsic therapeutic properties (e.g., photothermal therapy)	Often require functionalization with drugs or therapeutic agents
Stability	High stability under physiological conditions	Stability can vary depending on the material
Toxicity	Potential cytotoxicity if not properly designed	Generally lower intrinsic toxicity
Size and shape control	Precise control over size and shape for optimized performance	Good control, but may be more complex depending on the material
Surface modification	Easy surface modification with various ligands and targeting agents	Versatile surface modification, but may require complex chemistry
Biocompatibility	Varies, can be engineered for better biocompatibility	Generally good, but highly dependent on material and functionalization
Drug loading capacity	High capacity for drug loading and controlled release	Varies, generally high with polymer-based NPs
Heat generation	Effective in hyperthermia treatment (e.g., gold NPs)	Limited, primarily used in drug delivery rather than hyperthermia
Cost	Often more expensive due to material and synthesis methods	Typically, less expensive
Environmental impact	Potential environmental concerns if not properly disposed	Less environmental impact, biodegradable options available

This review aims to thoroughly explore the use of metal NPs in cancer therapy. This study will specifically focus on understanding the mechanisms behind the selective accumulation of metal NPs in specific locations. There are two ways to achieve this: one is by exploiting the permeable blood vessels found within tumors and the other is by specifically targeting receptors on cell surfaces. The aim of this work is to provide a comprehensive review about metal-based NPs for cancer therapy. This work will conduct a comprehensive analysis of the advantages and disadvantages associated with both non-noble and noble metals. It will evaluate the efficacy of metallic NPs in combating animal tumor and *in vitro* cancer cell lines to assess their clinical potential. Furthermore, the potential toxicity concerns of metal NPs in clinical applications will be addressed in the concluding section. Through the examination of these essential aspects, this review aims to augment the current understanding of metal NPs in cancer therapy and offer guidance for future research in this field.

## 2 Mechanism of delivery to tumors

**FIGURE 1:**
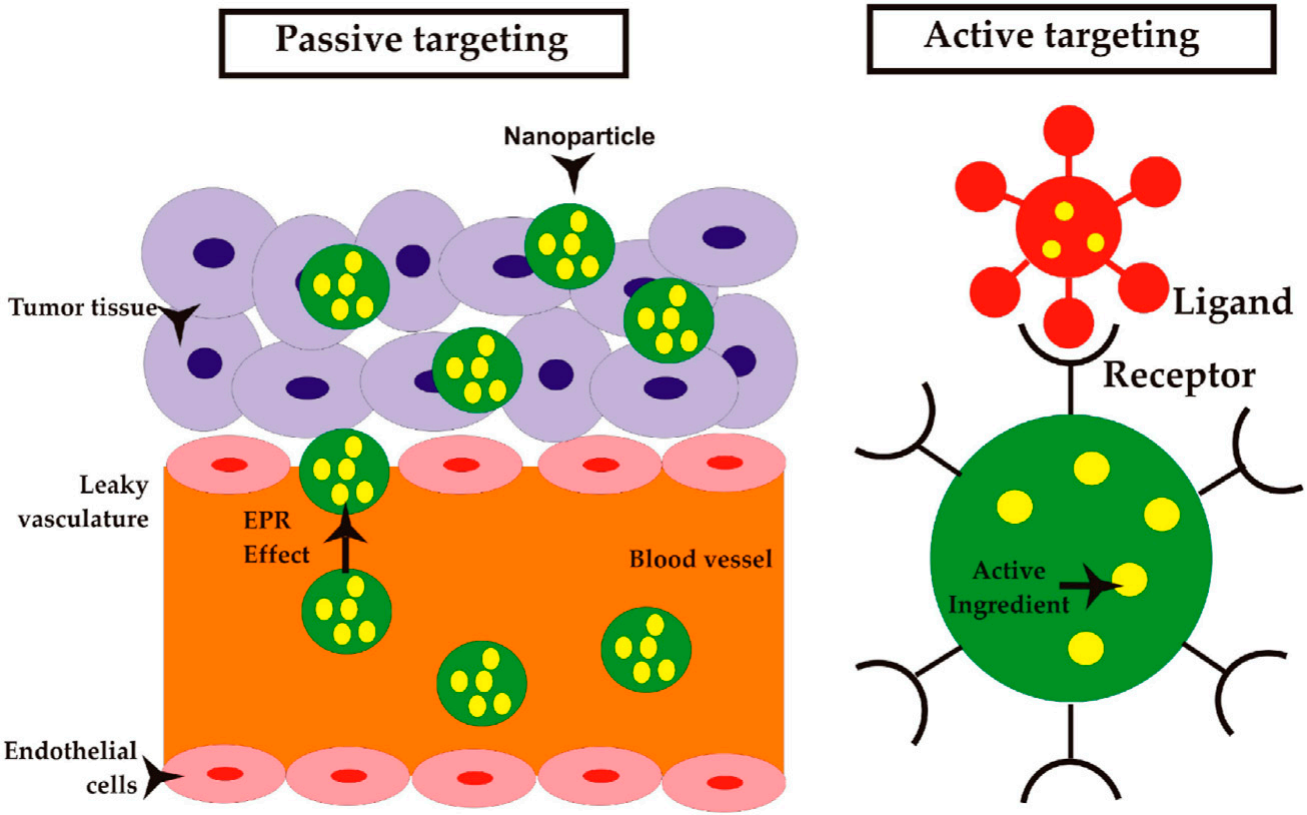
Mechanisms of NP delivery to solid tumors; passive targeting: NPs can be transported through the gaps between endothelial cells into blood microvessels, known as paracellular transport, as part of a passive targeting mechanism. Active targeting: NPs that are coated with ligands exhibit a transport mechanism that is analogous to that of NPs targeted passively. In contrast to NPs that employ passive targeting mechanisms, the utilization of ligand-coated NPs enables selective interactions with tumor cells, hence potentially enhancing NP retention and facilitating improved cellular uptake (Sevastre et al., 2019). Reproduced with permission from Sevastre et al. (2019). Copyright, Multidisciplinary Digital Publishing Institute, 2019.

There are two primary mechanisms that contribute to the accumulation of NPs within tumors: passive targeting and active targeting (Kashkooli et al., 2020; Li and Kataoka, 2020). The passive targeting method, also known as enhanced permeability and retention (EPR) effect, is a key strategy utilized by NPs to enhance their bioavailability and accumulation in tumor tissues. Active targeting is a process to enhance internalization through the modification of NPs via ligand deposition on their surface. Figure 1 shows a schematic view of these mechanisms, and Table 2 presents a summary of these mechanisms with their respective properties (Kashkooli et al., 2020; Kashkooli et al., 2021).

**TABLE 2:** Summary of delivery mechanisms of NP to solid tumors.

Classification criteria	Foundation of mechanism		Important factors influencing
Passive targeting	Accumulation of NPs through enhanced vascular permeability in solid tumors		Increasing accumulation of NPs in the interstitium
Active targeting	NP delivery utilizing ligand–receptor interactions	Antibodies: antibodies and their fragments are frequently used for precise delivery because of their exceptional specificity and affinity. Nevertheless, the potential safety concerns associated with these advancements present notable obstacles that could limit their use in clinical settings.	- High specificity and affinity - Good permeability and solubility - Low immunogenicity - Endogenous targets - Good penetration - Ease of synthesis - Manipulation potential
		Aptamers: aptamers exhibit greater permeability, solubility, and lower immunogenicity than antibodies. Their efficiency is affected by high clearance rates, rapid degradation by nucleases, ionic strength, temperature, and pH.	
		Small molecules: one of the key benefits of small molecules is their compatibility with straightforward pre-formulation conjugation techniques and the creation of customizable NPs. However, one of the main hurdles is finding new affinity ligands for desired substrates when using small molecules in NPs.	
		Peptides: peptides have a crucial role in immune response pathways, protein trafficking, and cellular signaling because of great contribution of peptide–protein interactions.	

### 2.1 Passive

In the context of tumor vasculature, the intercellular space between endothelial cells can range from 100 to 700 nm. By contrast, in healthy tissues, this space is typically limited to a maximum of 10 nm (Haley and Frenkel, 2008; Din et al., 2017). Furthermore, the lymphatic arteries exhibit limited functionality in tumors, resulting in insufficient reabsorption of molecules into the bloodstream. Consequently, this leads to a buildup of NPs at the location of the tumor. This scenario presents a highly effective strategy for implementing passive targeting. NPs exhibit a propensity to accumulate specifically within tumor tissue through the EPR effect, as elucidated before (Bertrand et al., 2014). The EPR effect is commonly observed in the majority of human cancers, with the exception of hypovascular tumors like prostate and pancreatic tumors (Maeda et al., 2009). It has been widely recognized as a benchmark and reference for all nanomedicine medications, enabling the use of NP administration in diverse scenarios to leverage this phenomenon (Danhier et al., 2010). The efficacy of EPR is maximized when the NPs exhibit prolonged circulation by evading immune surveillance. This action results in an elevation of drug NP concentrations by a factor of 10–50 compared to the levels observed in healthy tissue, namely, within the tumor region, for a duration of 1–2 days (Iyer et al., 2006). In order to accomplish this objective, it is crucial to take into account three specific qualities that hold significant relevance. (*i*) In order to efficiently eliminate NPs from the openings in permeable blood arteries, it is imperative that the NPs possess a size smaller than 400 nm. In order to circumvent renal filtration, it is imperative for particles to possess a size above 10 nm, while simultaneously maintaining a size below 100 nm, to prevent targeted sequestration by the liver. (*ii*) In order to efficiently evade renal filtration, it is important for the charge of the substances to either be neutral or anionic. (*iii*) In order to avoid elimination, it is necessary to conceal NPs from the reticuloendothelial system, which possesses the ability to eliminate any particles that it recognizes as alien entities through the process of opsonization followed by phagocytosis (Gullotti and Yeo, 2009; Malam et al., 2009; Danhier et al., 2010; Souri et al., 2024a).

However, despite the advantages of passive targeting through the EPR effect, there are certain limitations to consider. The effectiveness of passive targeting relies on factors such as microvessel density and angiogenesis, leading to variable extravasation of nanocarriers depending on the specific tumor type and anatomical location (Bae, 2009). Moreover, solid tumors often exhibit high interstitial fluid pressure, which can impede the cellular uptake of drugs and result in an uneven distribution within tumors. Typically, larger NPs (with a radius exceeding 100 nm) have a tendency to stay in tumors for longer periods, while smaller molecules show higher diffusivity (Pirollo and Chang, 2008; He et al., 2019). The presence of NPs in solid tumors is affected by the concentration of these carriers in the bloodstream, which in turn is influenced by various factors. Therefore, it is crucial to have a comprehensive understanding of the pharmacokinetics (PKs) of the drug in order to optimize passive targeting strategies (Soltani et al., 2021a; Souri et al., 2023). It is important to acknowledge these limitations associated with passive targeting in order to develop more effective strategies for NP-based drug delivery. By addressing these challenges, researchers can enhance the therapeutic outcomes and improve the overall efficacy of cancer treatments.

### 2.2 Active

Passive targeting leads to an increased concentration of NPs at the tumor site, but it does not enhance uptake by cancer cells. Consequently, active targeting has emerged as a strategy to facilitate specific interactions between NPs and cells, thereby improving the potential for NP uptake. The process usually involves using specific ligands on the surface of NPs to target receptors or other proteins on cancer cells (Table 3) (Dancy et al., 2020). Molecular components can engage in ligand–receptor interactions when they are in close proximity, usually within a distance of less than 0.5 nm (Hirsjarvi et al., 2011; Gao et al., 2020). Following extravasation and blood circulation, the contact between the ligand and receptor triggers receptor-mediated endocytosis, facilitating intracellular localization (Dhanasekaran and Chopra, 2016). The utilization of ligand-based NPs for active targeting has been recognized as a beneficial approach for drug delivery. Consequently, a multitude of specific ligands have been employed for the purpose of actively targeting NPs. A diverse array of synthetic and natural chemicals from many chemical families are employed as ligands to selectively target NPs against cancer cells (Ganipineni et al., 2018). Targeted ligands, which include antibodies, small molecules, aptamers, and peptides, play a crucial role in biomedical research. Choosing the right ligand is crucial for maximizing the effectiveness of NPs.

**TABLE 3:** Summarizing various types of ligands used in metal NP systems for cancer therapy, along with their reported efficiency in animal models.

Metal NPs	Ligand type	Ligand	Cancer type	Animal model	Efficiency	Ref.
AuNPs	Antibodies	Herceptin (trastuzumab)	Breast cancer (HER2+)	Mice	Selective targeting of HER2+ cells; significant tumor regression	Sitia et al. (2022)
AgNPs	Peptides	RGD peptide (Arg-Gly-Asp)	Melanoma	Mice	Inhibition of tumor growth; enhanced apoptosis	Fu et al. (2016)
Iron oxide NPs	Small molecules	Folic acid	Ovarian cancer	Mice	Targeted delivery; reduced tumor size	Krais et al. (2014)
AuNPs	Aptamers	AS1411	Lung cancer	Mice	High specificity; suppressed tumor growth	Yang et al. (2021)
Zinc oxide NPs	Polysaccharides	Chitosan	Colon cancer	Rats	Reduced tumor volume; enhanced immune response	Sharifi et al. (2023)
Copper NPs	Small molecules	Doxorubicin	Liver cancer	Rats	Improved drug delivery; significant tumor reduction	Xu et al. (2020)
AuNPs	Peptides	TAT peptide (trans-activator of transcription)	Brain cancer	Mice	Enhanced penetration; improved survival rate	Tiwari et al. (2014)
Iron oxide NPs	Polysaccharides	Hyaluronic acid	Prostate cancer	Mice	Effective targeting; decreased tumor mass	Nie et al. (2022)
AgNPs	Natural compounds	Curcumin	Multiple types	Mice	Anti-tumor and anti-inflammatory effects	Murugesan et al. (2019)
Platinum NPs	PEGylated lipids	PEG (polyethylene glycol)	Pancreatic cancer	Mice	Enhanced drug delivery; increased apoptosis	Boztepe et al. (2021)
AuNPs	Aptamers	Sgc8c	Leukemia	Mice	Specific targeting of cancer cells; reduced tumor burden	Khademi et al. (2023)

## 3 Mechanism of cancer therapy

Metal-based NPs have gained significant attention as potential candidates for cancer therapy due to their distinctive characteristics and wide range of applications. This section explores the diverse applications of metal-based NPs in combating cancer, such as their utilization as drug carriers and their involvement in necrosis and immunotherapy.

### 3.1 Drug delivery

In clinical practice, cancer medications often include small molecules that can easily enter both healthy cells and cancerous tissues. As a result, these findings indicate a wide distribution among the organisms and a fast rate of elimination. Reduced effectiveness in treatment and increased risk of negative effects, such as drug resistance, result from the limited amount of medications that reach the desired location (Ganipineni et al., 2018; Farzin et al., 2020; Abbasi et al., 2023; Gorgzadeh et al., 2023; Souri et al., 2024b). NPs with their high surface area provide vast sites for drug carrying, leading to enhanced stability and solubility of the loaded pharmaceuticals (Bilia et al., 2019). In addition, the use of targeted ligands to functionalize NPs has been proven to enhance the effectiveness of medications and minimize any potential negative effects (Elzoghby et al., 2020). Moreover, NPs have the advantage of engaging in multiple interactions with the cancer cell surface. In addition, NPs demonstrate improved PKs and more efficient accumulation in tumor tissues when compared to free medicines (Moradi Kashkooli et al., 2023; Shahvandi et al., 2023; Souri et al., 2024c). Finally, nanoscale pharmaceuticals exhibit a remarkable degree of biological selectivity, enabling them to selectively concentrate at tumor locations primarily as a result of the EPR effect (Zi et al., 2022). Although the delivery of therapeutic agents by NPs involves navigating various biological barriers (Table 4) (Wen et al., 2023), metal NPs have the ability to serve as carriers for precise and targeted transportation of therapeutic drugs. They can serve as carriers for both hydrophobic medications, such as paclitaxel-loaded selenium NPs and also doxorubicin (DOX) hydrophilic drug-loaded iron oxide (Fe_3_O_4_@SiO_2_@mSiO_2_) NP drug delivery systems (Bidkar et al., 2017; Gao et al., 2018). The enhanced bioavailability of pharmaceuticals is attributed to the tiny size and stability of the metal NPs. A study documented the development of a metal NP composition comprising a central core of superparamagnetic iron oxide, which was then coated with polyethylene glycol (PEG) of both short and long chain lengths. A metallic core was used to connect folic acid and paclitaxel, while the hydrophilic outer layer consisted of PEG. The NP system showed the ability to deliver drugs in response to a condition mimicking the acidic intracellular pH found in breast cancer cells, as compared to free paclitaxel. In addition, the NP’ folate conjugation resulted in increased uptake by target cells, thereby boosting toxicity toward these particular cells. In an independent study, researchers developed a chitosan/palladium nanocomposite to investigate its potential for simultaneous delivery of 5-fluorouracil and curcumin (CUR) to the colon. These drugs were loaded into the nanocomposite both separately and in combination. The co-encapsulated nanocomposite exhibits a stronger inhibitory effect on the proliferation of HT-29 cells than 5-FU or CUR used as monotherapy. Certain NP systems have greater IC_50_ values than unbound drugs due to the gradual release of the drug. However, these systems demonstrate satisfactory efficacy as targeted drug delivery methods. Table 5 presents a comparison of the IC_50_ values for metal NPs containing anticancer medications, and a comparison with the IC_50_ values of the corresponding free pharmaceuticals.

**TABLE 4:** Outlining the key barriers of drug delivery for metal NPs in cancer treatment: segmented from the blood, through tissue, to the cellular level.

Barrier level	Key barriers	Description
Blood	Plasma protein binding	Forming protein corona: a layer of proteins that forms on the surface of NPs, leading to rapid clearance from the bloodstream.
	Reticuloendothelial system (RES) uptake	RES, which includes the liver and spleen, can recognize and remove NPs from circulation.
	Vascular permeability	Limited permeability of blood vessels can restrict NP entry into tumor tissue.
	Hemorrheological properties	Blood flow dynamics can affect the distribution and accumulation of NPs.
	Immune system recognition	The immune system can recognize and eliminate foreign NPs.
Tissue	Extracellular matrix (ECM) density	Dense ECM can hinder NP penetration into tumor tissue.
	Interstitial fluid pressure (IFP)	Elevated IFP in tumors can oppose NP movement into the tumor.
	Tumor microenvironment heterogeneity	Variability in tumor vasculature and cellular composition can affect NP distribution.
	Enzymatic degradation	Enzymes in the tumor microenvironment can degrade or alter NPs.
Cellular	Cellular uptake mechanisms	Efficiency of endocytosis and other uptake pathways can vary among different cell types.
	Endosomal escape	After internalization, NPs must escape the endosome to release their payload.
	Intracellular trafficking	NPs must navigate intracellular compartments to reach their target site.
	Nuclear delivery	For certain therapies, NPs must cross the nuclear membrane, which is highly selective.
	Drug resistance mechanisms	Cancer cells may have mechanisms to resist NP-mediated drug delivery.

**TABLE 5:** The ratio of IC_50_ values between metal NPs containing anticancer drugs and their free drug counterparts.

Metallic NP	Anticancer drug	Cancer cell line	IC_50_ (NP/free drug)	Ref.
Copper NPs	Paclitaxel	Prostate cancer cell	0.075	Chen et al. (2018)
AuNPs	Doxorubicin	Glioma carcinoma cell	0.65	Mohanty et al. (2014)
AgNPs	Methotrexate	Breast cancer cells	0.51	Muhammad et al. (2016)
AuNPs	Chloroquine	Breast cancer cells	∼<1	Joshi et al. (2012)
AgNPs	Acetylshikonin (AS) and beta-dimethylacrylshikonin (BDS)	Human chronic myeloid leukemia	0.2	Ahmed et al. (2016)

**FIGURE 2:**
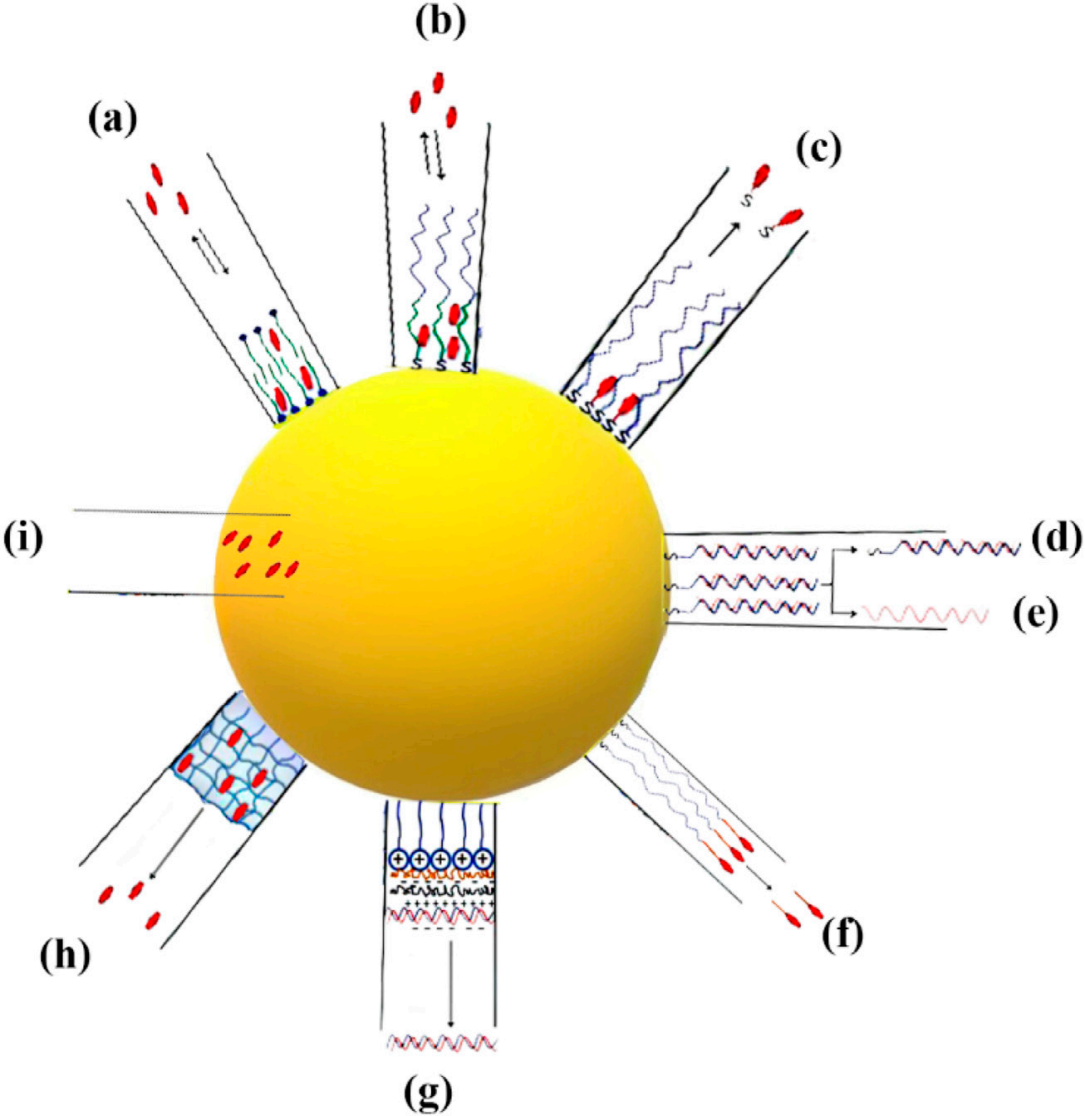
The illustration showcases several methodologies employed for the loading and unloading of therapeutic agents into and from metallic NPs. **(A, B)** The two options for the structure are either a bilayer composed of surfactant molecules or a layer consisting of amphiphilic molecules forming a corona. **(C)** The process of attaching pharmaceuticals to NPs by forming bonds NP-S or NP-N. **(D–E)** The utilization of NPs loaded with double-stranded (dsDNA) by the formation of bonds NP-S. The regulation of DNA release, whether in dsDNA or single-stranded DNA (ssDNA) form. **(F)** The capping agent’s terminal functional groups are used as attachment sites for therapeutic drugs through a cleavable linker. **(G)** Charged biomolecules are loaded onto the surfaces of AuNPs using the electrostatic assembly process. **(H)** Drug molecules are incorporated into a crosslinked thermosensitive polymer structure. **(I)** The encapsulation of drugs within NPs.

Typically, pharmaceutical substances can be encapsulated within or attached onto metallic NPs by using several techniques. In the following, drug loading methods explored by various studies are introduced, with a particular emphasis on those involving AuNPs (Figure 2):

- Loading by partitioning (Figures 2A, B): metal NPs such as AuNPs typically possess a monolayer or bilayer of a capping agent in their as-prepared state. This capping agent plays a crucial role as a stabilizer, preventing the NPs from aggregating. In certain cases, the capping agent can also play a role in shaping the NPs by influencing their growth. The presence of a mono- or bilayer can be advantageous in facilitating the loading and subsequent release of medications at a specific location, owing to a range of factors. One way to think about it is that metal NPs are surrounded by a thin layer of an organic solvent. This layer has the ability to selectively separate hydrophobic medicines from the surrounding environment (Alkilany et al., 2008). As an illustration, gold nanorods that have been created possess a surfactant bilayer on their surfaces, specifically cetyltrimethylammonium bromide (CTAB), with a thickness measuring approximately 3 nm. The study conducted by Alkilany et al. (2008) demonstrated the successful partitioning of hydrophobic compounds, specifically 1-naphthol, into the CTAB bilayer. The ratio of naphthol to CTAB on the surface of gold nanorods was found to be 1.6:1. Kim et al. (2009) created spherical AuNPs that were covered with a polymer layer. This polymer had both hydrophobic and hydrophilic regions, with the hydrophobic part on the inside and the hydrophilic part on the outside. The hydrophobic section of the polymeric shell of the NP was employed for the encapsulation of hydrophobic pharmaceutical compounds, while the hydrophilic portion was utilized to enhance the stability of the NPs in aqueous environments. This design demonstrates the capability of these NPs to sequester hydrophobic medicines and subsequently release them upon interaction with the cell membrane, obviating the necessity for internalization of the NPs within the cell. Until the NPs interacted with cells, in the aqueous solutions, the payload remained stable and prevented the release of cargo. Based on these findings, it was found that the mechanism of release within the cellular membrane is because of the transportation of drugs to the hydrophobic regions from the polymer monolayer (Kim et al., 2009).
- Loading by surface complexation (Figures 2C–E): the rationale behind this loading strategy stems from the strong attraction between thiols and amines and metal surfaces (Murphy et al., 2010). Pharmaceutical compounds containing thiols or free amines, either as inherent constituents or introduced without altering the inherent activity of the medicine, have the capability to attach to AuNPs by means of Au-S or Au-N bonding interactions (Dreaden et al., 2009; Murphy et al., 2010). The aforementioned methodology has been employed for the purpose of conjugating pharmaceutical compounds, small interfering RNA (siRNA), and DNA onto the exterior of AuNPs (Jain et al., 2006; Cheng et al., 2008; Papp et al., 2010; Poon et al., 2010). The release process can be started in response to various stimuli. It is important to acknowledge in this context that the manner in which drugs form complexes with the gold surface has an impact on their release profile. In the context of pharmaceuticals containing thiol groups, the bonding between gold and sulfur atoms (Au-S bond) has sufficient strength to impede drug release through passive diffusion (Jain et al., 2006). Complex therapies that include Au-S bonds frequently require the assistance of extrinsic stimuli, such as external light or thiol exchange, for their release. In the context of amines, it is observed that the strength of the Au-N bond is comparatively lower than that of the Au-S bond. This disparity in bond strength can potentially offer a favorable condition for enhanced drug release through the process of diffusion, hence facilitating improved drug delivery efficiency (Cheng et al., 2010). In their study, Cheng et al. (2010) observed distinct delivery profiles of photodynamic treatment (PDT) in cancer medications when these chemicals were linked to the gold surface through either Au-N or Au-S bonds. In the first scenario, the strong interaction between the drugs and NP core hindered the release process. On the other hand, in the second situation, there was a noticeable enhancement in the release profiles in both the two-phase system (water:toluene) and intracellular environments. The researchers discovered that weak contacts between NPs and drugs are more advantageous than covalent bonding with the NP surface. Surface complexation was found to be a useful method for attaching or releasing pharmaceuticals from the surface of NPs, offering the convenience of monitoring loading and release events through fluorescence microscopy. Fluorescence quenching occurs when a fluorophore is connected to an NP surface, like a gold core (Dulkeith et al., 2002; Sapsford et al., 2006). Observing changes in fluorescence intensity can provide valuable insights into the monitoring of drug loading and its kinetics.
- Loading by attachment to capping agents (Figure 2F): therapeutic drugs can be combined with AuNPs using either complexation or by attaching them to the functional groups of the capping agents. In these instances, the gold surface has already undergone passivation by the introduction of diverse functional groups. Subsequently, drug attachment occurs on the topmost layer, situated atop the particles. Wheate et al. utilized carboxylic acid moieties on AuNPs to create complexes with platinum-based anticancer drugs. Additionally, they employed this approach to synthesize platinum-tethered AuNPs, with the aim of inducing cytotoxicity in lung and colon cancer cells (Brown et al., 2010). In their study, Dhar et al. (2009) employed a method that involved the utilization of single-stranded DNA on gold nanospheres. This allowed for the connection of platinum-based prodrugs with carboxylic acid groups, resulting in the formation of amide bonds. These prodrug-AuNPs could effectively penetrate cancer cells, leading to the conversion of the platinum core from Pt(IV) to Pt(II). This reduction process facilitated the release of cisplatin, a potent anti-cancer medication. Rothrock et al. employed a method wherein nitric oxide (NO) donor molecules were affixed to the terminal amines located on AuNPs, resulting in the generation of gold nanospheres capable of releasing NO. This novel approach has promise for future utilization in vasodilation-related endeavors (Rothrock et al., 2005; Polizzi et al., 2007). AuNPs were utilized as carriers in a study conducted by Agasti et al. (2009) to attach an additional anticancer drug, specifically 5-fluorouracil. The attachment process involved utilizing carboxylic acids obtained from the capping agents of the NPs. These acids were then connected to the drug using a photosensitive o-nitrobenzyl linkage. Drug release was seen as a result of the breakage of the photosensitive linker upon exposure to UV light. In this context, it is imperative to emphasize a significant observation pertaining to the conjugation of medicinal drugs with the capping agents on the gold surface. Coupling processes frequently result in NP aggregation, particularly when the medicines being coupled have hydrophobic properties. As an illustration, Gibson et al. (2007) synthesized gold nanospheres with a diameter of 2 nm, featuring a compact paclitaxel shell. To achieve this, researchers modified paclitaxel by incorporating a flexible hexaethylene glycol linker. Subsequently, the carboxylic acid of the linker was attached to AuNPs that were terminated with phenol groups. The organic shells of paclitaxel enclosed within the attachment were found to constitute 67% of the total weight. However, it is worth noting that the inclusion of the paclitaxel organic shell around the NPs led to a substantial decrease in their solubility in aqueous solutions (Gibson et al., 2007).
-Loading by using layer-by-layer assembly (Figures 2G, H): AuNPs show a significant level of charge because of charged capping agents on NP surfaces. Taking this into consideration, it is possible to effectively bind charged pharmaceuticals to the gold surfaces that possess complementary charges using electrostatic conjugation or the associated layer-by-layer (LbL) coating technique (Gole and Murphy, 2005; Murphy et al., 2010). An excellent example of this loading technique involves the association of nucleic acids with AuNPs through charge complexation (Huang et al., 2009; Chakravarthy et al., 2010). To enhance gene delivery and suppress gene expression, DNA and siRNA molecules, which are characterized by their negative charge, can be effectively combined with cationic AuNPs (Caruso et al., 1998). It is noteworthy to mention that the LbL technique involving complementary charged polymers results in a highly robust contact, frequently characterized by irreversibility. The association of nucleic acids, such as DNA or siRNA, with AuNPs can potentially hinder the release of the payload. To overcome this challenge, Guo et al. (2010) utilized a charge-reversal co-polymer that could adjust the zeta potential in response to pH changes. This approach aimed to address the issue of payload retention. At neutral pH conditions, the charge of the polymer is predominantly negative, enabling it to form complexes with cationic AuNPs. However, in acidic environments like endo/lysosomes, the particle acquires an overall positive charge. As a result, this change in charge facilitates the detachment of cationic NPs from the surface, thereby releasing the previously bound DNA or RNA. In a study by Lee et al. (2011), the LbL technique was utilized to coat AuNPs with multiple layers of siRNA (three layers) and poly-L-lysine (four layers). This approach effectively prevented the aggregation of the NPs. An advantageous property of poly-L-lysine is its biodegradability, particularly its susceptibility to protease activity. This unique characteristic allows for the continuous release of the siRNA that is complexed with poly-L-lysine, resulting in a prolonged effect of gene silencing.
- Loading inside the NPs (Figure 2I): hollow gold nanoshells and gold nanocages, which are hollow metal nanostructures, possess advantageous characteristics for drug delivery applications. These include their significant overall surface area and the presence of interior reservoirs that can be used for loading therapeutics (Moon et al., 2011; Yang et al., 2011). In their study, Yavuz et al. (2009) developed gold nanocages, which are nanostructures composed of porous hollow gold with unique optical and photothermal properties. These characteristics make them highly capable of absorbing and scattering light in the NIR range of the electromagnetic spectrum. The researchers utilized these gold nanocages to design an advanced drug delivery system that allows for controlled release of medication. They achieved this by encapsulating drug molecules within the hollow interior of the nanocages and coating them with a dense thermosensitive polymer on the outer surface. The presence of the polymer shell effectively prevents the release of the drug unless triggered by thermal stimuli. Gold nanocages have demonstrated excellent efficacy in capturing near-infrared (NIR) photons, facilitating the release of payloads. This release mechanism involves the absorption of NIR light, which is then converted into heat. The generated heat causes the thermosensitive polymer shell to melt, exposing the pores in the nanocage walls and resulting in the release of the encapsulated medicine. The smart polymers are attached to the outer surface of the nanocages through gold–thiol bonds, while the drug was loaded into the interior of the nanocages through inward diffusion from aqueous fluids. By manipulating the laser power density and exposure period, the release profile can be precisely controlled, preserving the integrity of the nanocubes and preventing the polymeric shell from detaching. This example demonstrates the superiority of AuNPs over other NPs, like polymeric NPs, when it comes to light-induced release in various scenarios. AuNPs offer a distinct advantage over non-metallic NPs that rely on ultraviolet (UV) light for cleaving a photosensitive organic linker. This advantage lies in the ability of AuNPs to adjust the wavelengths at which they absorb light. This property allows for their utilization in the NIR range, where minimal light absorption and scattering occur in biological components and tissue (Alvarez-Lorenzo et al., 2009).

### 3.2 Hyperthermia

Significant alterations, such as cellular death, transpire when biological substances experience a slight elevation in temperature beyond their typical range. Hyperthermia therapy is a therapeutic modality employed in the treatment of cancer, wherein elevated temperatures are applied to bodily tissues, often ranging from 40 to 45°C (113°F), with the intention of inducing detrimental effects or the death of cancerous cells (Jose et al., 2020). The primary objective of hyperthermia-related occurrences is to induce alterations in the extracellular environment through the activation of immune responses and the induction of a shift in tumor cells toward an anerobic metabolic system (Balivada et al., 2010; Hosseinpour et al., 2024). There are three different types of hyperthermia, each with its own specific area of application: whole-body hyperthermia, localized hyperthermia, and regional hyperthermia. The extent of invasiveness or noninvasiveness in whole-body hyperthermia depends on the particular method used for application. There are two types of hyperthermia procedures: invasive and noninvasive. Invasive hyperthermia involves externally heating the blood, while noninvasive hyperthermia utilizes methods like hot wax, hot air, or radiofrequency or infrared irradiation to increase the temperature. It is important to consider that noninvasive hyperthermia may not be effective for treating malignancies that are located deep within the body (Wust et al., 2002). Regional hyperthermia is a targeted therapeutic approach that aims to induce hyperthermia in a specific area. This can be accomplished through noninvasive methods, such as the use of NIR or ultrasound to heat tumors located in the desired region. Invasive techniques, such as thermal conduction or the use of magnetic implants, can also be utilized (Longo et al., 2016). The aforementioned treatments, whether invasive or noninvasive, are employed to increase the temperature of small tumors up to a depth of 4 cm in the localized hyperthermia method (Jose et al., 2020). The field of hyperthermia has experienced significant advancements, largely attributed to the emergence of nanotechnology, particularly magnetic NPs. Exploring the heat dissipation mechanisms exhibited by these NPs can provide valuable insights into the underlying process of tumor eradication through hyperthermia. Magnetic NPs enhance the transfer of thermal energy to tumor cells through two distinct mechanisms: Néel relaxation and Brownian relaxation. Néel relaxation is a fascinating phenomenon where the magnetic moment aligns itself parallel to the applied magnetic field. On the other hand, Brownian relaxation occurs when the nanomaterial undergoes mechanical rotation in response to the external magnetic field. When a medium is exposed to an external alternating magnetic field (AMF) with a magnetic field reversal time shorter than the material’s magnetic relaxation period, both Néel and Brownian relaxation processes occur simultaneously (Chen et al., 2017; Patade et al., 2020; Souri et al., 2022c). The production of high-quality superparamagnetic MnFe_2_O_4_ NPs was carried out using a cost-effective and environmentally sustainable co-precipitation method, with the aim of utilizing them for hyperthermia applications. The findings of the study conducted by Patade et al. (2020) have demonstrated that MnFe_2_O_4_ magnetic NPs are capable of achieving hyperthermia temperature (42°C) within a time frame of 260 s, even at a low concentration of 0.4 g/mL. These results suggest that the material holds potential for use as a heating agent in magnetic hyperthermic treatment. The study conducted by Ma et al. (2019) documented the synthesis of Fe_3_O_4_-Pd Janus NPs (JNPs) that exhibit amplified dual-mode hyperthermia and enhanced reactive oxygen species (ROS) formation, hence demonstrating potential for breast cancer treatment. When subjected to a combination of AMF and laser irradiation, Fe_3_O_4_-Pd JNPs demonstrated a greater increase in temperature compared to when either AMF or laser irradiation was applied individually to Fe_3_O_4_-Pd JNPs. Additionally, the temperature enhancement achieved by the combined modality was higher than the sum of the temperature enhancements achieved by the two separate modalities. In the acidic environment, the presence of H_2_O_2_ led to an increase in the creation of ROS by Fe_3_O_4_-Pd JNPs. This increase can be attributed to the synergistic impact at the interface, where the Fe_3_O_4_ NPs facilitate the Fenton reaction and the Pd nanosheets exhibit catalytic capabilities. As a result of this interface synergy, hydroxyl radicals (OH) are created. Remarkably, the application of external AMF in conjunction with laser irradiation resulted in an even more pronounced elevation of the ROS level. In order to evaluate the efficacy of Fe_3_O_4_-Pd JNPs in tumor treatment, experiments were conducted on mice with orthotopic breast cancer. The combined use of Fe_3_O_4_-Pd JNPs and laser irradiation, guided by MRI/photoacoustic (PA) dual-mode imaging, has demonstrated remarkable spatial resolution and precision. This approach resulted in complete tumor suppression while minimizing any significant adverse effects (Ma et al., 2019).

**FIGURE 3:**
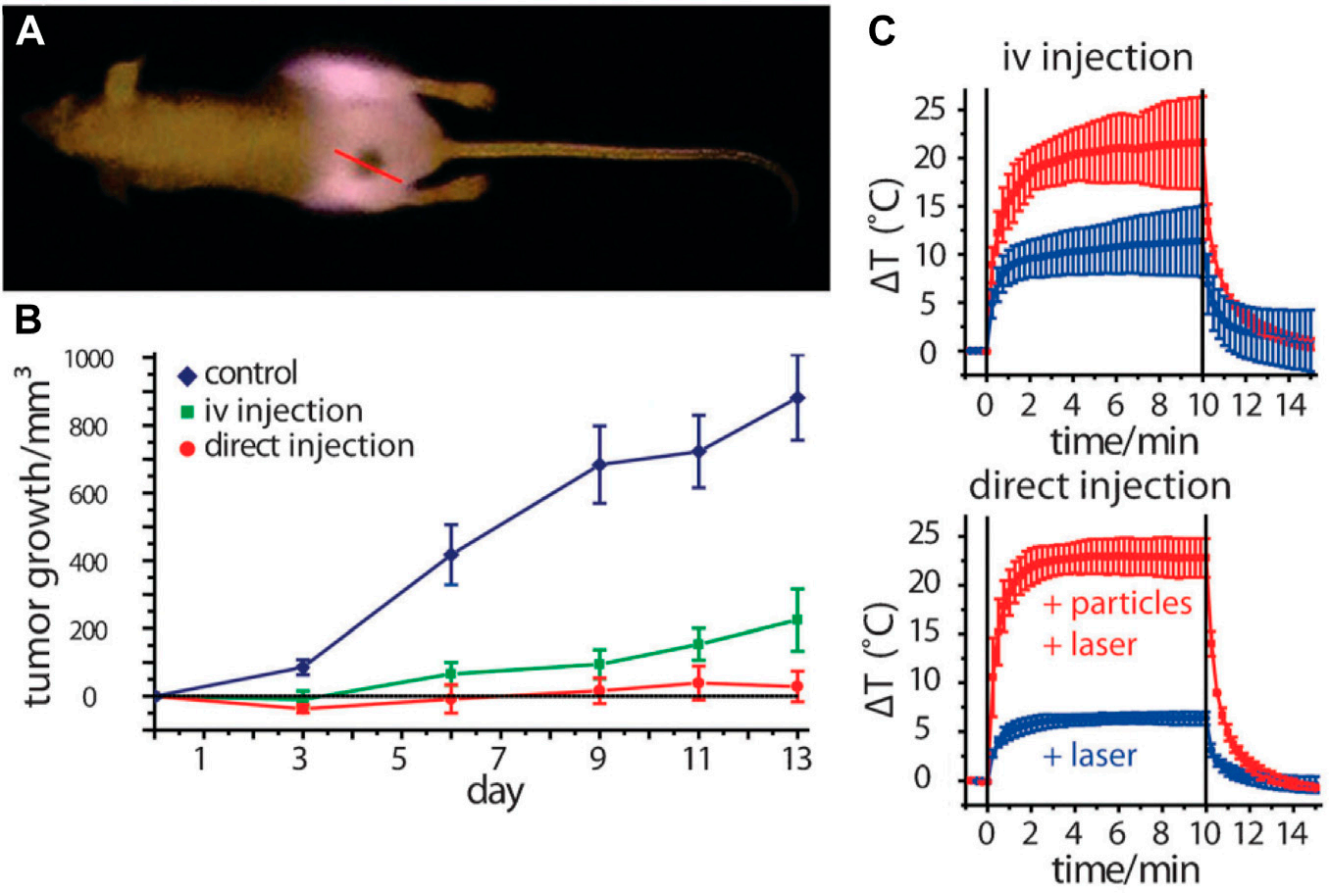
Utilizing gold nanorod contrast agents in laser photothermal therapy for cancer treatment: **(A)** pre-treatment of NIR transmission images of mice before undergoing plasmonic photothermal therapy, **(B)** quantify the average change in tumor volume for HSC-3 xenografts following NIR plasmonic photothermal therapy treatment using three different administration methods, **(C)** analysis of thermal transient measurements in the interstitium of HSC-3 tumors during direct and intravenous NIR photothermal therapy treatment (Dickerson et al., 2008). Reproduced with permission from Dickerson et al. (2008). Copyright, Elsevier, 2008.

AuNPs have emerged as a prominent tool in the field of medicine, specifically in the context of photothermal therapy (Lal et al., 2008; Norman et al., 2008). The power of AuNPs to effectively absorb light and transform it into thermal energy is a subject of great interest. This unique characteristic has been utilized in various applications, such as the eradication of cancer cells, germs, and viruses (as discussed later in this text). Hence, AuNPs that have been subjected to laser radiation have the potential to function as therapeutic agents independently, obviating the necessity for co-conjugated pharmaceutical compounds. AuNPs exhibit a notable capacity for light absorption, characterized by high efficiency, as indicated by their extinction coefficient of B109 M^−1^cm^−1^ (Orendorff et al., 2006). The utilization of NIR radiation enables the implementation of photothermal therapy at significant tissue depths, owing to the enhanced light penetration capabilities exhibited by NIR wavelengths (Weissleder, 2001). AuNPs and classical photosensitizers exhibit distinct mechanisms of action in the context of PDT. AuNPs possess the ability to generate heat upon exposure to irradiation, whereas classical photosensitizers primarily produce singlet oxygen. This notable distinction highlights the diverse ways in which these two types of agents contribute to the effectiveness of PDT (Huang et al., 2008). Both of these modalities effectively contribute to the eradication of undesirable cells throughout the therapeutic process. AuNPs have several advantageous characteristics in comparison to other NPs. The favorable attributes of AuNPs, such as efficient absorption, enhanced solubility, and the ability to easily conjugate with targeted molecules and medicines, make them highly advantageous for the implementation of photothermal therapy in the treatment of cancer and other pathogenic disorders. These distinctive characteristics position AuNPs as promising candidates for advancing the field of therapeutic applications. The application of gold/silica nanoshells in photothermal therapy was pioneered by Lal et al. (2008). Nanoshells are nanostructures that consist of a thin gold shell and a silica core. These structures possess the unique property of adjustable optical extinction, allowing them to exhibit varying levels of absorption and scattering of light within the visible to NIR range. The degree of optical attenuation is dependent on the dimensions of both the core and shell. Nanoshells have been utilized to ablate various malignant cell lines in laboratory settings and have shown effectiveness in treating cancer in animal models through *in vivo* experiments when exposed to NIR irradiation. This highlights the potential of nanoshells as a promising approach for cancer treatment. Despite the apparent simplicity of nanoshell manufacturing and their advantageous plasmonic properties, it is crucial to acknowledge that these particles are considerably larger in size (around 130 nm) than other AuNPs that absorb NIR light. This size reduction could potentially impede the aggregation of nanoshells within specific malignant cells or restrict their elimination from the body. Nonetheless, Nanospectra Biosciences is presently engaged in conducting FDA-sanctioned human pilot studies for AuraLases, a product that harnesses the potential of gold nanoshells. This underscores the ongoing efforts to explore and optimize the use of nanoshells in clinical applications (Schwartz et al., 2009). Gold nanorods, which have been produced by Murphy and El-Sayed (Dickerson et al., 2008), exhibit great potential as viable options in the field of plasmonic phototherapeutics. Gold nanorods possess several advantageous characteristics. First, they are relatively simple to synthesize. Additionally, their plasmonic absorbance may be adjusted to desired levels. In addition, gold nanorods are typically smaller in size than gold–silica nanoshells. These nanorods have shown promising effectiveness in ablating colon cancer and squamous cell carcinoma. The pioneering work of Jain et al. (2008) established the use of gold nanorods for photothermal therapy. The researchers presented compelling evidence demonstrating the efficacy of this approach in suppressing tumor growth. Remarkably, in certain cases, a single laser exposure lasting only 10 min resulted in complete resorption of the tumor. These findings highlight the potential of utilizing gold nanorods for targeted photothermal therapy, offering a promising avenue for effective tumor treatment (Figure 3).

### 3.3 Radiotherapy

Radiation treatment employs high-energy radiations to impede the proliferation or induce the death of cancerous cells (Piranfar et al., 2024). Ionizing radiation is the principal focus of concern in the context of cancer therapy. Ionizing radiation is a form of electromagnetic radiation that possesses enough energy to induce ionization, a process where electrons are stripped away from atoms or molecules, leading to the formation of ions. These ions, which possess significant kinetic energy, undergo multiple collisions, leading to the transfer of a substantial amount of energy to the cells they pass through. The energy that is delivered is adequate to inhibit the replication of DNA or the transcription of RNA in tumor cells, leading to cellular demise (Ebrahimi Fard et al., 2017; Song et al., 2017; Carozza et al., 2020). One of the most challenging aspects of radiotherapy (RT) involves the precise administration of a lethal dose of radiation to target tumor cells, while minimizing the risk of inadvertent harm to surrounding healthy cells. Metal NPs are extensively utilized in the field of RT to enhance the selectivity of radiation toward the intended site, hence diminishing the radiation dosage and mitigating the potential harm and toxicity to healthy tissues (Igaz et al., 2020; Schuemann et al., 2020). The phenomenon of ionizing radiation induces the process of radiolysis in water molecules, leading to the generation of ROS. The presence of an unpaired electron renders them capable of inducing substantial DNA damage. Metal NPs employ many strategies to enhance the specificity of radiation targeting. Metals have been found to enhance oxidative stress in tumor cells, facilitate preferential apoptosis, and diminish clonogenic survival (Choi et al., 2020; Igaz et al., 2020; Schuemann et al., 2020). In recent research on radiation therapy, several types of metal NPs have been employed. AgNPs and AuNPs have shown exceptional performance compared to other metal NPs in applications involving cancer imaging and therapy, particularly in radio sensitization. The combination of ionization and hyperthermia has proven to be an effective therapeutic approach for cancer management. The concept of employing simultaneous treatment to induce hyperthermia in tumor cells while concurrently administering radiation therapy is seemingly ideal. However, it is widely believed that hyperthermia administered prior to radiation therapy yields greater success rates. Inverse metal NPs have been found to enhance the precision of radiation targeting, while concurrently inducing a hyperthermic reaction specifically at the site of the tumor. The combination of heat, metal, and room temperature in cancer treatment has been observed to result in a notable increase in response rates, ranging from 16% to 26% (Cędrowska et al., 2020; Tolkaeva et al., 2021).

Sears et al. have demonstrated the sensitivity of triple-negative breast cancer to both ionizing radiation and photothermal therapy (Sears et al., 2021). AgNPs with a dominant absorbance peak in the NIR range were synthesized. In their study, the researchers aimed to selectively treat MDA-MB-231 triple-negative breast cancer cells while minimizing harm to nonmalignant MCF-10A breast cells. To achieve this, they utilized a multimodal approach that combined ionizing radiation sensitization, photothermal therapy, and targeted cytotoxicity. The experimental findings show that the implementation of triangular AgNPs for thermal radiation sensitization yielded remarkable outcomes, even when employing a lower treatment dose and frequency. These findings highlight the efficacy of triangular AgNPs in enhancing thermal radiation sensitization, despite the reduced dosage and frequency of treatment (Sears et al., 2021). The experimental investigation involved the examination of the potential synergistic effects of combining AuNPs with the histone deacetylase inhibitor suberoylanilide hydroxamic acid (SAHA), as well as their impact on radio sensitization. This investigation was conducted using both two-dimensional (2D) and three-dimensional (3D) cancer cell cultures. The efficiency of the treatment was assessed using A549 and DU-145 cancer cells that showed resistance to radiation. The administration of AuNPs and SAHA before RT resulted in a substantial decrease in the viability of cells. This observation suggests that the concurrent use of AuNPs and SAHA considerably enhanced the effectiveness of irradiation (Igaz et al., 2020).

Neutron capture therapy (NCT) is another promising cancer treatment modality that utilizes the nuclear capture reactions of non-radioactive isotopes like boron-10 (^10^B) or gadolinium-157 (^157^Gd) with low-energy neutrons to generate high-energy particles that can selectively damage tumor cells. Metal NPs have emerged as potential delivery vehicles for these isotopes, offering several advantages over conventional small-molecule agents (Table 6) (Vitale et al., 2005; Sauerwein et al., 2012).

**TABLE 6:** Advantages, disadvantages, and mechanism of neutron capture therapy (NCT) using metal NPs for cancer treatment.

Aspect	Details
Mechanism	- NCT mechanism: involves the nuclear capture reactions of non-radioactive isotopes like boron-10 (^10^B) or gadolinium-157 (^157^Gd) with low-energy neutrons. - Particle generation: these reactions generate high-energy particles (alpha particles for ^10^B and gamma rays for ^157^Gd ) that selectively damage tumor cells. - Metal nanoparticles role: metal NPs act as delivery vehicles for these isotopes, enhancing their accumulation in tumor cells and improving treatment efficacy.
Advantages	- Selective damage: high-energy particles generated specifically damage tumor cells, minimizing harm to the surrounding healthy tissue. - Enhanced delivery: metal NPs improve the delivery and retention of isotopes in tumor cells. - Versatility: metal NPs can be engineered to target specific tumor types and improve treatment specificity. - Reduced side effects: compared to traditional therapies, NCT can have fewer side effects due to its targeted nature. - Synergy with other treatments: can be combined with other therapies (e.g., chemotherapy and radiotherapy) for enhanced efficacy.
Disadvantages	- Complexity: requires precise delivery of NPs and accurate targeting of tumor cells. - Neutron source requirement: access to a suitable neutron source is necessary, which can be limited and expensive. - Dosimetry challenges: accurate dosimetry is crucial to ensure effective treatment while avoiding damage to healthy tissue. - Limited penetration: the penetration depth of generated particles can be limited, affecting treatment of larger or deeper tumors. - Safety concerns: handling and delivery of radioactive materials and NPs require stringent safety measures.

*Boron-based metal NPs for BNCT*: Boron NCT (BNCT) relies on the ^10^B(n,α)^7^Li nuclear reaction, where the α particles and lithium nuclei produced have a short range (<10 μm) and can selectively kill cells containing sufficient amounts of ^10^B (>20 μg/g tumor tissue) (Heide et al., 2021; Ailuno et al., 2022). (*i*) Iron–boron (Fe-B) NPs have been developed by coating iron oxide NPs with boron compounds like carboranes. The Fe component allows magnetic targeting and MRI tracking, while boron provides the therapeutic effect (Ailuno et al., 2022; Oloo et al., 2023). (*ii*) Bimetallic Fe-Gd-B NPs combine the benefits of BNCT and gadolinium neutron capture therapy (GdNCT), enabling simultaneous MRI and synergistic tumor cell killing (Ailuno et al., 2022; Shanmugam et al., 2023). (*iii*) Boron-rich protein nanotubes have been explored as carriers, offering high boron loading capacity and cellular uptake (Heide et al., 2021).

*Gadolinium-based metal NPs for GdNCT*: In GdNCT, the ^157^Gd(n,γ)^158^Gd reaction generates Auger electrons and gamma rays that can penetrate deeper into tissues than can BNCT particles. (*i*) Anti-EGFR-targeted Gd10B6 NPs delivered high amounts of ^10^B (158 μg/g tumor) and ^157^Gd (56.8 μg/g tumor) to head and neck tumors in mice, enabling effective combined GdBNCT with long survival times (Shanmugam et al., 2023).

### 3.4 Photodynamic therapy

PDT is an emerging technique that is now being explored as a potential solution to address the requirement for a precise cancer treatment that could potentially decrease the likelihood of cancer recurrence and prolong patient survival while minimizing adverse effects (Wilson, 2002; Gunaydin et al., 2021). The healing properties of visible light have been recognized since ancient times. Several clinical studies have been conducted, which include phase III, to investigate the efficacy of PDT (Stummer et al., 2006; Stepp et al., 2007; Stummer et al., 2008). These studies have employed various technologies, such as interstitial PDT (iPDT) and surgical PDT (Pinel et al., 2019). The utilization of interstitial PDT presents a targeted therapeutic strategy that may lead to enhanced management of glioblastoma, potentially resulting in substantial increases in patient survival rates (Pinel et al., 2019).

The optimization of PDT modalities necessitates consideration of multiple phenomena associated with one or more primary components, namely, the photosensitizer, light, and oxygen, which play crucial roles in determining therapeutic efficacy (Van Straten et al., 2017). The nonlinear interactions associated with dosimetry provide a significant challenge (Romano et al., 2022). The extent to which light can penetrate into the target tissue is contingent upon its distinct optical qualities. In the event that the tissue experiences hypoxia or undergoes hypoxia due to treatment, it is anticipated that the production of singlet oxygen O_2_ will be less than the anticipated levels (Allison and Moghissi, 2013). In addition to the aforementioned complexities, it is important to note that the concentration of the photosensitizer, extent of light penetration, and level of tissue oxygenation may exhibit variability throughout the course of treatment, with each parameter potentially exerting an influence on the others. The utilization of NPs in PDT represents a significant advancement in addressing the various difficulties encountered with conventional photosensitizers (Sun et al., 2018). In their study, Wang et al. (2011) employed titanium dioxide-based NPs to investigate the efficacy of combining surgical resection with local PDT in mice with glioma. It is noteworthy that these NPs, which are biocompatible in nature, can be activated through photocatalysis using UVA radiation (Kubota et al., 1994; Lee et al., 2009). AuNPs have become promising candidates for effective drug administration due to their easy functionalization, superior surface chemistries, and adjustable size. In a study conducted by Dixit et al. (2015), a multifunctionalized NP was successfully engineered to enhance photosensitizer selectivity. This engineered NP enabled intraoperative PDT after fluorescence-guided resection, specifically targeting tumor cells.

**FIGURE 4:**
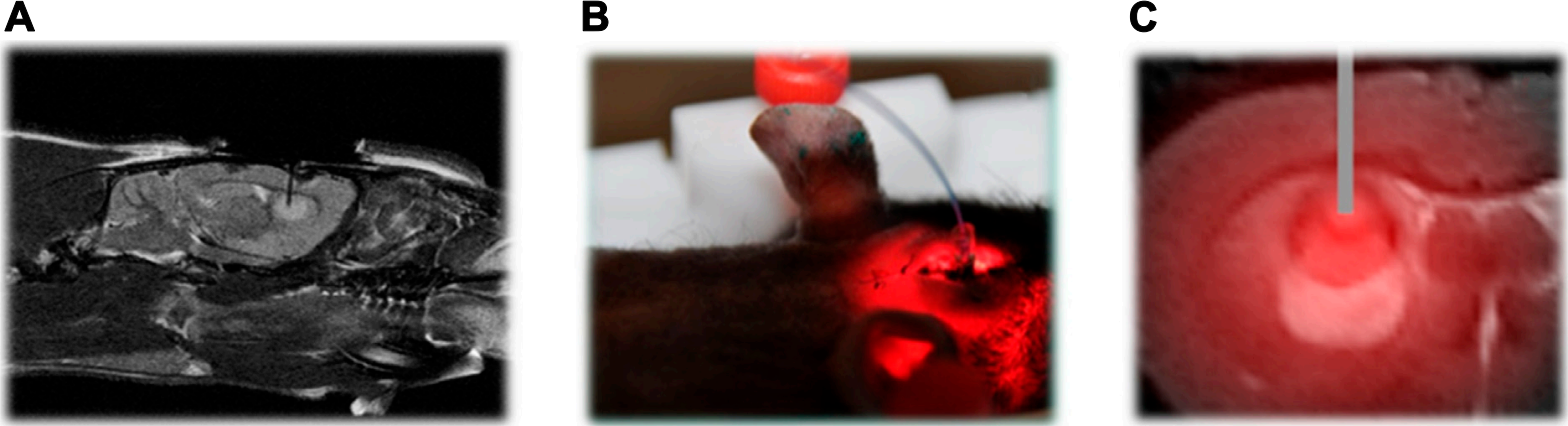
**(A)** Proton-weighted images of the fiber insertion in the sagittal plane. **(B)** To ensure precise positioning of the optical fiber into the brain tissue, a skull anchor was patented. **(C)** MRI analysis following intravenous AGuIX-porphyrin NP injection into rats with intracranial Glioblastoma Multiforme (GBM) and fiber placement shown in tumor tissue (Pinel et al., 2019). Reproduced with permission from Pinel et al. (2019). Copyright, Elsevier, 2019.

**FIGURE 5:**
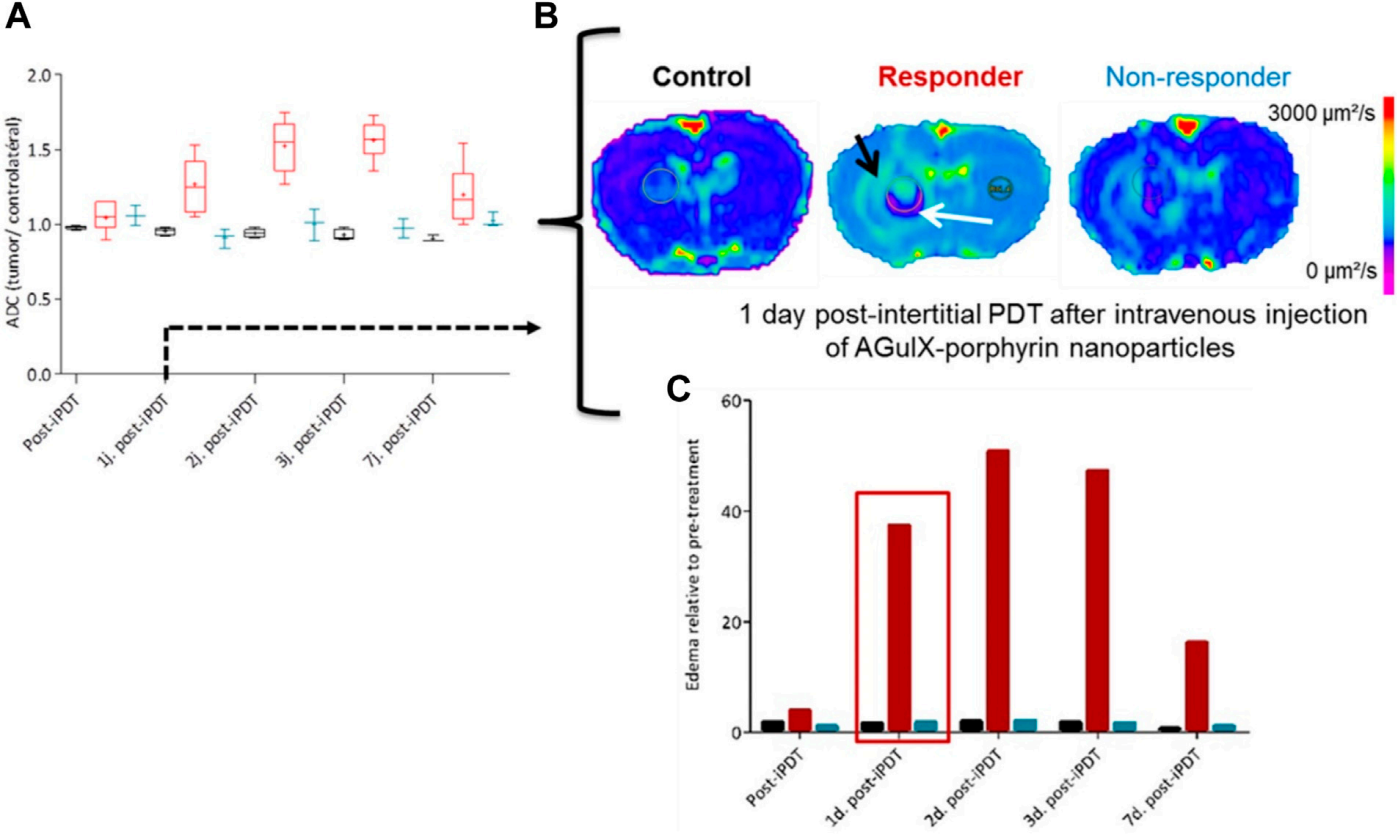
Following the completion of 1 day post iPDT **(A)**, the utilization of diffusion-weighted imaging **(B)** facilitated the identification of potential indicators, notably the apparent diffusion coefficient values **(C)** (Toussaint et al., 2017). Reproduced with permission from Toussaint et al. (2017). Copyright, Ivyspring International Publisher, 2017.

In the case of tumors that cannot be surgically removed, the utilization of several optical fibers to administer light is a potential approach for PDT. The field of noninvasive imaging in small animal research has made significant advancements in recent years. Recent advancements in technology have facilitated the rapid retrieval of highly accurate data, encompassing several levels of information such as morphological, functional, and molecular details (Pinel et al., 2019). One notable benefit of noninvasive imaging techniques is in their ability to incorporate a time dimension in the assessment of a biological reaction, allowing for the dynamic monitoring of its progression *in vivo*, particularly in longitudinal research. The integration of contrast agents with nanomedicine has made significant contributions to the field of noninvasive imaging, particularly in the context of cancer management. This coupling enables real-time monitoring of the drug’s bioavailability and therapeutic response, thereby providing vital support in cancer treatment. In the context of using iPDT to glioblastoma, MRI emerges as a suitable choice. In order to confirm the use of iPDT guided by MRI for glioblastoma, a patent was obtained for a skull anchor device that facilitates precise control over the placement of the optical fiber within the brain (Figure 4) (Pinel et al., 2019). The assessment of early indications pertaining to the effectiveness of PDT and the growth of tumors continues to be crucial in the characterization of photo-induced effects. The study conducted by Toussaint et al. demonstrated that the utilization of spectroscopic and diffusion MRI monitoring techniques can effectively forecast the tumor response following the application of iPDT. The authors employed an NP design known as AGuIX^®^, which exhibits multifunctionality (Toussaint et al., 2017). These NPs were formulated with gadolinium, enabling its usage in MRI. Additionally, the NPs were coupled with a porphyrin compound, serving as a photosensitizer. In this study, the impact of iPDT on glioblastoma was investigated. The researchers examined the apparent diffusion coefficient values and the expression levels of lipids, myo-inositol, and choline (Figure 5) to assess the effects of iPDT. These findings suggest that these parameters could serve as early noninvasive markers of treatment success (Toussaint et al., 2017).

### 3.5 Anti-angiogenic

The pivotal function of angiogenesis in various diseases, such as cancer, rheumatoid arthritis, and macular degeneration, is widely acknowledged (Dvorak, 2002; Ferrara and Kerbel, 2005; Ferrara et al., 2007). In normal physiological conditions, the process of angiogenesis is regulated through the interaction of various anti-angiogenic factors, such as TSP-1 and platelet factor 4, as well as pro-angiogenic growth factors like vascular endothelial growth factor (VEGF), transforming growth factor β (TGF-β), and PDGF (Tosetti et al., 2002). However, in pathological situations, the equilibrium is broken, leading to the activation of the angiogenic switch (Tosetti et al., 2002). This occurrence elicits the formation of significantly atypical blood vessels that exhibit heightened permeability to plasma proteins. Currently, certain anti-angiogenic medicines are being utilized in clinical settings; nevertheless, the bulk of these medications have been specifically developed to solely impede VEGF-mediated signaling (Yap et al., 2009). Furthermore, it has been reported that these conventional agents have demonstrated unforeseen and severe toxicities, such as hypertension, thrombosis, and deadly bleeding. Moreover, the available clinical evidence suggests that focusing on a singular pathway is not the optimal or efficacious approach to treatment (Bergers and Hanahan, 2008).

**FIGURE 6:**
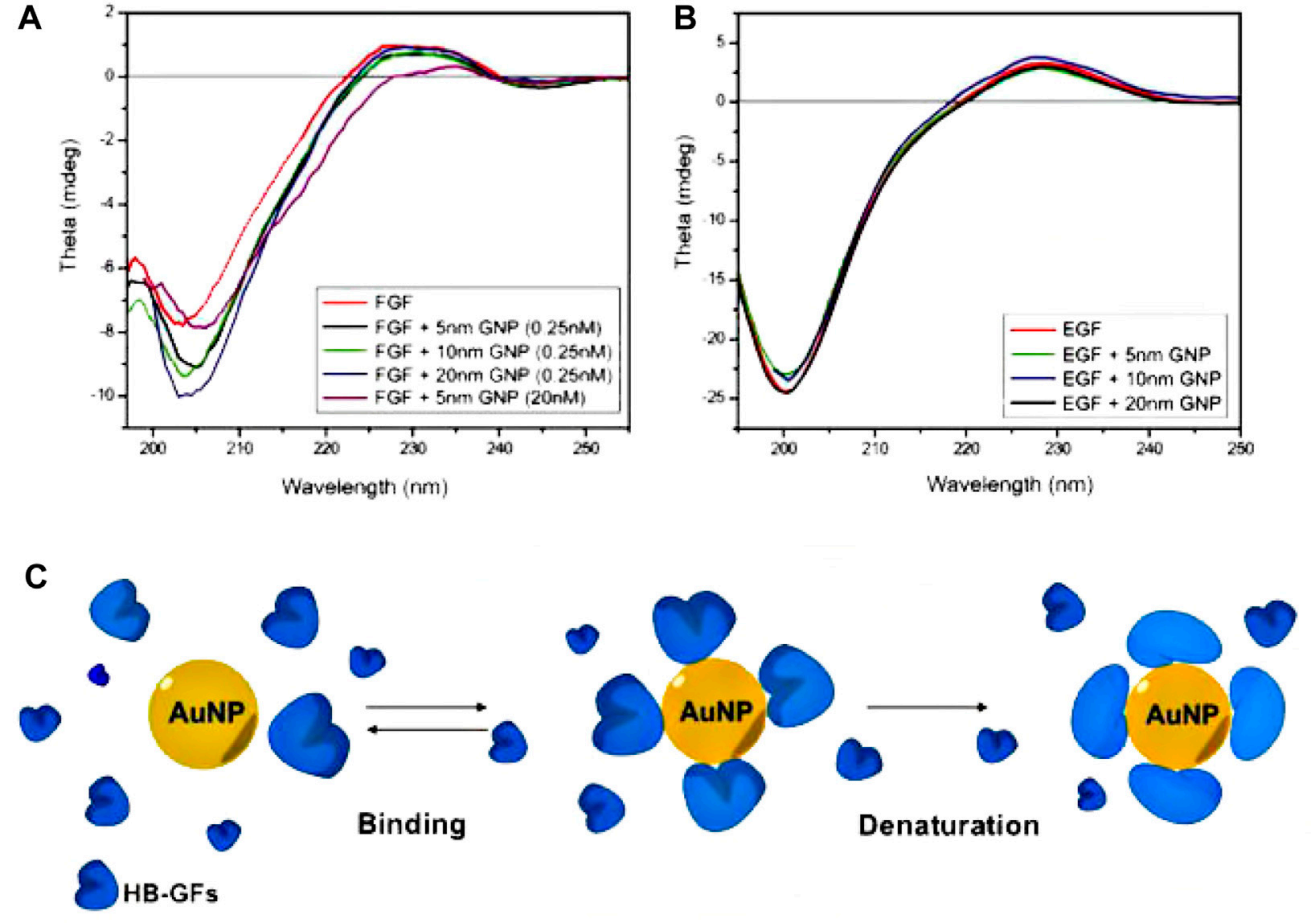
The interaction between AuNPs and heparin-binding growth factors (HB-GFs) has been observed to lead to the suppression of their biological activity. This effect is believed to be caused by changes in the conformation of the proteins. **(A, B)**. **(A)** A solution containing 0.2 mg/mL of basic fibroblast growth factor (bFGF) was subjected to incubation both with and without AuNPs in a 5 mM phosphate buffer. **(B)** A solution containing EGF at a concentration of 0.15 mg/mL was subjected to incubation both with and without Gold Nanoparticles (GNPs), using the same experimental procedures as previously described. The data sets were adjusted by subtracting the values corresponding to blank samples with the same concentration of AuNPs in the buffer. **(C)** The provided image depicts a visual representation of the process of protein denaturation occurring on the surface of AuNPs (Arvizo et al., 2011a). Reproduced with permission from Arvizo et al. (2011a). Copyright, Elsevier, 2011.

Due to the aforementioned considerations, it is plausible that metal NPs could potentially exhibit enhanced efficacy, as they have demonstrated the ability to selectively target numerous routes (Bergers and Hanahan, 2008). If these NPs demonstrate independent efficacy as anti-angiogenic agents, they could potentially address the unusual toxicities associated with traditional anti-angiogenic drugs. A notable study has revealed that “uncoated” AuNPs effectively suppressed the activity of heparin-binding proteins, such as bFGF and VEGF, in laboratory settings. Additionally, these NPs were observed to impede VEGF-induced angiogenesis *in vivo* (Mukherjee et al., 2005). Nevertheless, proteins that do not attach to heparin, such as VEGF and epidermal growth factor (EGF), maintained their inherent functionality. Subsequent investigations in this field have provided additional insights, revealing that proteins with heparin-binding properties adhere to the surface of AuNPs and undergo subsequent denaturation. The researchers had additionally demonstrated that the therapeutic impact of AuNPs is mostly influenced by surface size rather than surface charge (Bhattacharya et al., 2004; Arvizo et al., 2011a). In this research investigation, Arvizo et al. (2011a) conducted a preincubation of vascular with citrate-reduced AuNPs of varying diameters (5, 10, and 20 nm). The aim of the study was to evaluate the influence of these AuNPs on the signaling pathways in human umbilical vein endothelial cells. This assessment is illustrated in Figure 6 (Arvizo et al., 2011a). The presented results demonstrate that 20 nm citrate-reduced AuNPs had a significant effect on various VEGF signaling processes, which include proliferation, receptor-2 phosphorylation, and intracellular calcium release, in comparison to other conditions. In a study conducted by Mukherjee et al., the impact of AuNPs on VEGF-mediated angiogenesis was investigated using an *in vivo* model. This involved injecting an adenoviral vector of VEGF to simulate the angiogenic response observed in tumors. After 1 week, the mice treated with AuNPs exhibited reduced edema compared to those that received sham treatment (Arvizo et al., 2011a).

Furthermore, recent research conducted by Gurunathan et al. (2009) has demonstrated that AgNPs possess anti-angiogenic properties. In their study, 40-nm AgNPs were utilized to examine their effects on angiogenesis in bovine retinal epithelial cells. The experiments, which included both *in vitro* and *in vivo* experiments using a Matrigel plug, showed that the presence of AgNPs effectively inhibited cell proliferation and migration during VEGF-induced angiogenesis. This discovery suggests that AgNPs may have the ability to target and activate the PI3K/Akt signaling pathway (Gurunathan et al., 2009). The authors proceeded to disclose that the *in vivo* suppression of new blood vessel creation was observed in the presence of AgNPs. Additionally, the researchers conducted further investigations to elucidate the anti-tumor properties of 50-nm AgNPs both in laboratory settings (*in vitro*) and in living organisms (*in vivo*) (Sriram et al., 2010). A study was conducted to investigate the effects of AgNPs on Dalton’s lymphoma ascites (DLA) cell lines and tumor growth in mice. When the DLA cell lines were co-incubated with AgNPs, a dose-dependent toxicity was observed. This toxicity was characterized by the activation of caspase-3, a protein involved in cellular apoptosis, and a reduction in cellular proliferation. Furthermore, in mice with tumors that were injected with AgNPs, a significant decrease of 65% in the generation of ascites and slower tumor growth were observed than in mice that received sham treatment. These findings suggest that AgNPs have the potential as a therapeutic agent for lymphoma treatment (Sriram et al., 2010).

### 3.6 Gene silencing

Gene silencing is a regulatory mechanism used by cells to prevent the expression of specific genes. Gene silencing holds promise as a potential cancer therapy due to its ability to decrease the expression of genes that are linked to tumor formation. Gene silencing refers to the modulation of gene expression at an epigenetic level. The predominant approach to achieving this is through the use of antisense short iRNA and DNA (Fernandes and Baptista, 2017; Liu et al., 2019; Souri et al., 2022d). Fernandes and Baptista (2017) conducted a study whereby they utilized multifunctional AuNPs to achieve gene silencing for the purpose of enhancing tumor cell identification and uptake in the context of cancer therapy. The surfaces of AuNPs were subjected to functionalization by the use of targeting peptides. The aforementioned methodology effectively suppresses the expression of the KRAS gene in cell lines of colorectal cancer, while exhibiting no detrimental effects on healthy fibroblast cells. Another potential approach is the utilization of siRNA to induce gene silencing. Researchers utilized a metal–organic framework (MOF) coated with a membrane of platelet cell to deliver siRNAs into cells. They successfully loaded synthetic siRNAs onto porous MOF NPs using a simple one-pot method, resulting in effective delivery. The stability of MOF scaffolds was found to be pH-dependent. The study focused on human SK-BR-3 breast cancer cells and conducted *in vitro* analysis to target and locate NPs within the cells (Fernandes and Baptista, 2017).

### 3.7 Cancer immunotherapy

The control over physicochemical properties of metallic NPs makes them highly useful for cancer immunotherapy applications (Niikura et al., 2013; Salatin et al., 2015; Soltani et al., 2021b). In contrast to nanoformulations composed of non-metallic materials of comparable sizes, metallic NPs with higher densities exhibit enhanced cellular uptake, hence conferring an advantage for cancer vaccination approaches (Barnaby et al., 2014). Metallic NPs possess unique optical characteristics that can be effectively utilized in the context of metallic NP-mediated tumor ablation in conjunction with immunotherapy (Arvizo et al., 2012; Almeida et al., 2014). This section will provide an overview of the diverse range of techniques, applications, and preclinical achievements observed in metallic NP immunotherapies.

**FIGURE 7:**
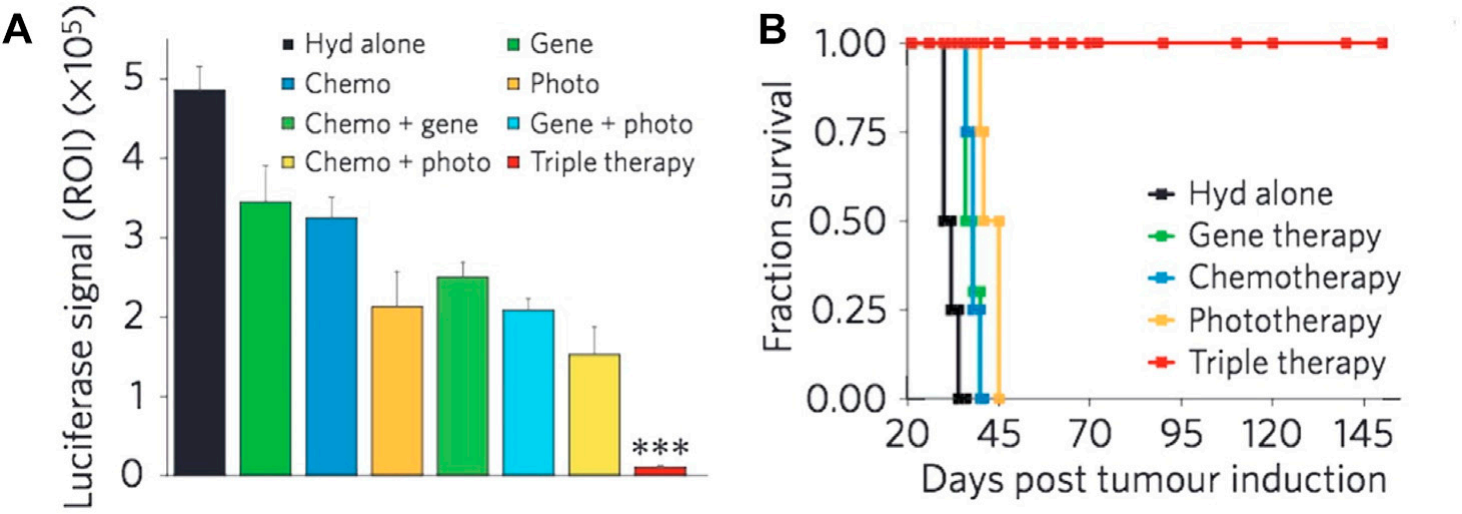
The concurrent administration of chemotherapy, siRNA, and photothermal therapy resulted in a significant reduction in tumor burden **(A)** and a notable improvement in survival rates when compared to the individual administration of each therapy **(B)** (Evans et al., 2018). Reproduced with permission from Evans et al. (2018). Copyright, Elsevier, 2017.

**FIGURE 8:**
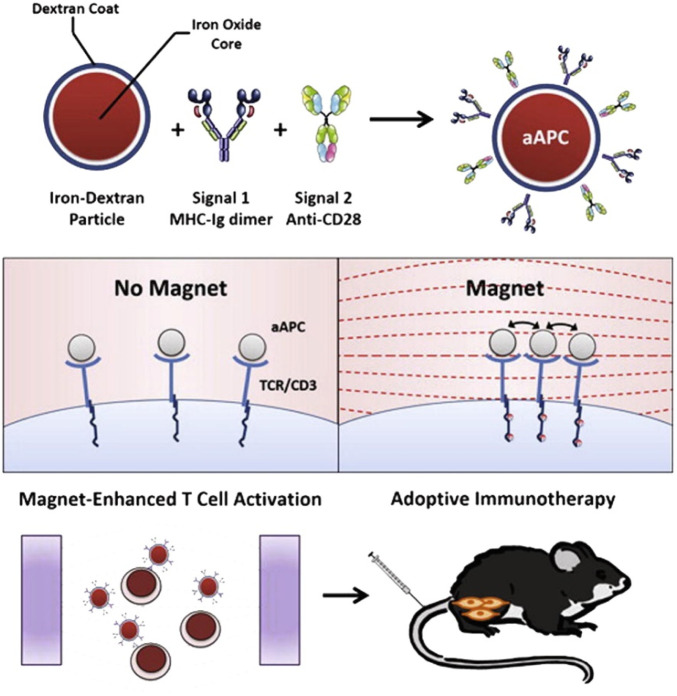
The clustering of TCRs with paramagnetic NPs resulted in an enhanced *ex vivo* growth of T cells and showed greater efficacy in adoptive T-cell therapy for melanoma tumors (Perica et al., 2014). Reproduced with permission from Perica et al. (2014). Copyright, American Chemical Society, 2017.

(i) The enhancement of antigen and adjuvant delivery: the use of metallic NPs has been shown to enhance vaccine delivery by promoting the uptake of antigens by dendritic cells (DCs) and other antigen-presenting cells. This, in turn, leads to an improved cytotoxic T-cell response against tumors (Ilyas and Yang, 2015; Schumacher and Schreiber, 2015; Shao et al., 2015). In an early instance of this occurrence, Chen et al. (2010) employed AuNPs of different sizes to administer antigens and documented substantial serum antibody responses toward the administered antigen. Subsequent studies have utilized AuNP platforms for the delivery of tumor-associated antigens, frequently showcasing proof-of-concept achievements through the utilization of ovalbumin (OVA) as a representative antigen. Ahn et al. (2014) conducted a study wherein they provided evidence that AuNPs effectively transport OVA to DCs, hence promoting cross-presentation and subsequently impeding the progression of tumor growth. The *in vivo* experiment demonstrated that AuNPs coated with peptides induced a humoral response, as seen by the observed augmentation in IgG production. This immune response was facilitated by the blimp/pax5 pathway (Lee et al., 2014). Almeida et al. (2015) conducted a study showing that delivering OVA antigens using AuNPs significantly improved efficacy in reducing tumor burden and enhancing survival rates. This effect was observed in both therapeutic administrations and prophylactic. By contrast, administering OVA alone did not result in an immune response or provide any survival benefits.
(ii) Utilizing optical properties for enhancing immunotherapy: certain research groups employ optical properties exhibited by metallic NPs to investigate the underlying principles of tumor biology and the field of cancer immunotherapy (Biju et al., 2008; Evans et al., 2018; Bai et al., 2020). The aforementioned mechanistic data can be utilized in the development of more effective therapeutic interventions. An illustration of this may be seen in the study conducted by Yang et al. (2017), where AuNPs and mass cytometry were employed to detect immune cells at the single-cell level. The findings of this research shed light on the advantages of surface modification of metallic NPs, which led to enhanced particle absorption. The AuNPs, upon undergoing this alteration, effectively facilitated the delivery of OVA antigens to DCs, resulting in successful vaccination and subsequent decrease of tumors in an *in vivo* setting. Furthermore, the utilization of noninvasive approaches such as metallic NPs for *in vivo* tracking of immune cells holds promise for clinical application in assessing patient reactions to immunotherapies. Metallic NPs have been employed by many research groups to visualize immune cells *in vivo* using imaging techniques such as CT and MRI (Meir et al., 2015; Kirschbaum et al., 2016; Chhour et al., 2017). Recent literature studies have examined the utilization of metallic NPs in the context of diagnostic and monitoring applications, specifically focusing on their potential in cancer immunotherapy (Ahrens and Bulte, 2013; Liu et al., 2014; Meir et al., 2014; Lee et al., 2016; Kim et al., 2017). These reviews also address various opportunities and obstacles associated with the clinical translation of metallic NPs in this field.
(iii) Focusing on the immunological microenvironment of tumors: tumors are frequently unfavorable for the survival and proper functioning of immune cells (Gajewski et al., 2013). The diminished efficacy of cytotoxic T cells can be attributed to many factors such as the acidity levels in the surrounding environment, tumor signaling, and the presence of immune-suppressive cytokines (Frey, 2015). The utilization of metallic NPs has been employed for the purpose of delivering chemicals that modify the microenvironment, hence creating a more conducive environment for immune cell infiltration and subsequent detection and eradication of tumor cells (Duan et al., 2016a). The study found that when gold nanoshell-mediated photothermal therapy and gene therapy were combined, NF-κβ signaling was downregulated specifically at the tumor site. This downregulation reduced the tumor’s pro-tumorigenic effects caused by the transcription factor and also increased the tumor’s responsiveness to subsequent chemotherapy (Lu et al., 2010). AuNPs were utilized as carriers to transport siRNA in a targeted manner, resulting in the specific suppression of VEGF production in both tumor cells and tumor-associated macrophages. This targeted gene silencing led to the regression of the tumor, as depicted in Figure 7 (Conde et al., 2015; Conde et al., 2016; Evans et al., 2018). Metallic NPs have exhibited effectiveness in selectively targeting regulatory T cells (Tregs), which are responsible for immune suppression, hence reducing the activity of immune cell pathways associated with suppression. The presence of cuprous oxide NPs modifies the expression of a *Drosophila* transcription factor, leading to the initiation of myeloid infiltration and subsequent systemic immunization (Yu et al., 2017).
(iv) Enhancing cell-based therapies (*ex-vivo*): due to the intricate nature of immune system initiation in living organisms, certain modalities of immunotherapy employ molecular biotechnology techniques to alter immune cells outside of the body (*ex vivo*) and subsequently reintroduce them to patients (Perica et al., 2015; Scholz et al., 2017). The utilization of NPs has the potential to enhance the effectiveness of *ex vivo* pulsed antigen-presenting cells, such as DCs and macrophages. The utilization of a nanoAu-cocktail consisting of AuNPs-OVA and AuNP-CpG resulted in enhanced immune protection against exogenous antigens through the activation of pulsed DCs (Zhou et al., 2016). The study conducted by Cho et al. (2011) provided evidence that DCs loaded with iron oxide/zinc oxide core–shell NPs exhibited a reduction in tumor size, an enhancement in survival rates, and an additional advantage of serving as an imaging contrast agent. *In vivo*, the augmentation of antigen-specific T-cell responses was seen upon the administration of cobalt oxide NPs to macrophages (Chattopadhyay et al., 2016). NPs possess the capability to mitigate certain constraints associated with adoptive T-cell treatment through the *ex vivo* delivery of materials. In a particular study, the utilization of iron oxide NPs resulted in the enhancement of T-cell proliferation and activation through the spatial aggregation of CD3 T-cell receptors (TCRs; as depicted in Figure 8) (Perica et al., 2014). In a separate study, Schütz et al. (2016) performed the conjugation of TCRs and major histocompatibility complex IgG (MHC-IgG) with magnetic NPs. This conjugation aimed to activate T cells *ex vivo*, which then led to a reduction in tumor burden when the modified T cells were administered *in vivo* to immunocompromised mice

**FIGURE 9:**
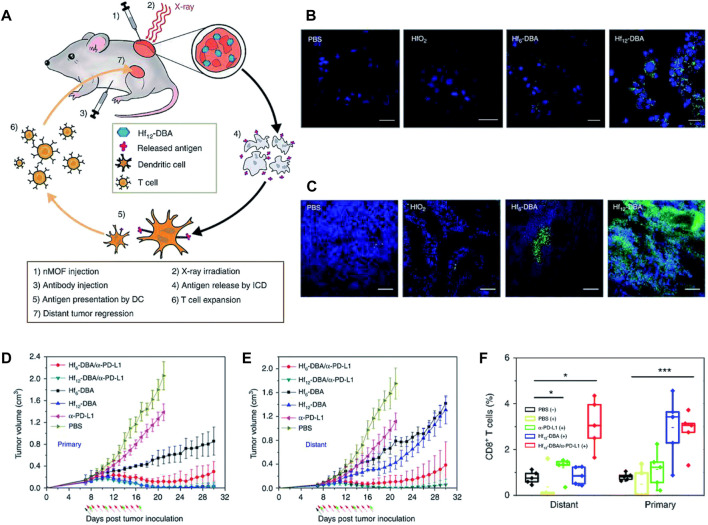
Metal–organic frameworks have emerged as promising candidates for augmenting the efficacy of radiation therapy and checkpoint blockade immunotherapy. **(A)** The abscopal effect of nanoporous MOFs in combination with radiation therapy and immune checkpoint inhibition. The expression of calreticulin is observed both **(B)** *in vitro* and **(C)** *in vivo*. The tumor development curves of **(D)** primary tumors and **(E)** distant tumors **(E)** in CT26 bilateral tumor-bearing mice were analyzed under various treatment conditions. **(F)** Tumor-infiltrating CD8^+^ T lymphocytes were observed in both the main tumors and distant malignancies (Ni et al., 2018). Reproduced with permission from Ni et al. (2018). Copyright, Nature, 2018.

The combination of RT and cancer immunotherapy has emerged as a significant treatment approach with promising outcomes in tumor ablation. This combined modality exploits the ability of immunotherapy to induce supplementary damage to tumors during RT-mediated local therapy, while also augmenting the effectiveness of RT in treating distant tumors. Checkpoint blockade immunotherapy is a highly promising strategy for stimulating antitumor immune responses (Smyth et al., 2001; Dunn et al., 2002). However, the dysregulated expression of immune-checkpoint proteins serves as a crucial mechanism of immunological resistance, effectively suppressing T-cell function within tumor microenvironments. There is a desire to utilize immunomodulatory adjuvant treatments in order to combat immune resistance and bolster the effectiveness of antitumor immunity (Pardoll, 2012; Tumeh et al., 2014). Recent empirical findings have demonstrated that the administration of large doses of ionizing radiation as an adjuvant treatment has the potential to induce an immunomodulatory response (Schaue, 2017; Zhang et al., 2019). Nevertheless, the current approach still faces several challenges. For instance, the administration of high-dose ionizing radiation can lead to significant damage to healthy tissue. Additionally, the immunomodulatory impact caused by RT poses a barrier to achieve widespread tumor rejection throughout the body. In recent times, there has been the utilization of nanomaterials containing heavy metals, coupled with antibodies and inhibitors, to address the aforementioned challenges. This approach serves the dual purpose of minimizing X-ray exposure while effectively targeting tumors, as well as augmenting the efficacy of checkpoint blockade immunotherapy in promoting systemic anticancer immune responses (Ni et al., 2018). Ni et al. (2018) presented their findings on the utilization of Hf-based MOFs in combination with the anti-programmed cell death ligand-1 antibody. These MOFs were found to possess radiosensitizing properties, leading to a significant enhancement of local RT effects. Furthermore, the authors observed that these effects could be transferred to distant tumors through the occurrence of abscopal effects, as depicted in Figure 9. In the context of local therapy, it was observed that Hf-based nano metal–organic frameworks (Hf6-DBA and Hf12-DBA) exhibited higher efficacy as radiosensitizers than did hafnium dioxide (HfO_2_). The assessment of the cellular presentation of calreticulin (CRT) on the surface of cells, both in laboratory settings (*in vitro*) and in living organisms (*in vivo*), confirmed that Hf12-DBA can induce a more robust form of cell death that triggers an immune response. This finding aligns with the observed release of HMGB1 from cells, suggesting that Hf12-DBA-mediated RT may possess the ability to kill cells through immune mechanisms. Furthermore, the findings regarding antitumor immunity suggest that the combination of MOF-mediated RT with PDL1 checkpoint blockade therapy exhibits a greater capacity to induce optimal antitumor efficacy in distant tumors compared to alternative treatment groups (Ni et al., 2018). The researchers observed an increase in the presence of NK cells and CD^8+^ T cells within the tumors, which contributed to the enhanced effectiveness. To achieve systemic eradication of tumors, they developed a novel approach that combines indoleamine 2,3-dioxygenase-loaded MOFs with localized low-dose RT and anti-PD-L1 antibody. The aforementioned methodologies, which involve the utilization of metal-based nanomaterials, demonstrate a compelling synergy between RT and immunotherapy. This synergy effectively enhances the efficacy of local RT while simultaneously reducing the adverse effects on healthy tissues. In addition, it has the capability to exhibit significant antitumor efficacy on remote cancers (Ni et al., 2018).

## 4 Metal-based nanoparticles in cancer therapy

In the field of cancer therapy, a wide variety of metallic NPs have been employed to great effect. Metal NPs can be classified into two categories: noble and non-noble metal-based NPs, which are manufactured using various processes (Table 7).

**TABLE 7:** The synthesis methodology employed for the production of the most prevalent metallic NPs utilized in cancer therapy.

NP	Synthesis method	Ref.
AuNPs	Chemical based: the synthesis of AuNPs through chemical means involves the reduction process of various oxidizing agents, such as HAuCl_4_, within a solution containing inositol hexakisphosphate (IP6), chloroauric acid, trisodium citrate, and thiolated chitosan.	Pérez et al. (2011), Saleh (2011), Amjadi and Farzampour (2014), Giljohann et al. (2020)
	Physical based: physical method is the utilization of γ-irradiation technique, which results in the production of AuNPs with a high level of purity and uniform size distribution ranging from 5 to 40 nm. This technique involves the incorporation of the polysaccharide alginate as a stabilizer throughout the synthesis process. The microwave irradiation approach was employed to synthesize AuNPs, employing several reducing agents such as CTAB and citric acid as a binding agent.	Rezende et al. (2010), Guo et al. (2012)
	Green approach: the production of AuNPs via a green approach involves the utilization of several biomolecules as substitutes for traditional stabilizing and reducing agents. These biomolecules can include green plants, algae, yeast, fungi, and bacteria. According to the paper, the reduction of HAuCl_4_ was achieved through the utilization of extracts derived from citrus fruit juice. Furthermore, the utilization of the edible mushroom in the synthesis of AuNPs was facilitated with the application of light energy.	Tarnawski and Ulbricht (2011), Nalawade et al. (2012)
AgNPs	Chemical-based: the successful chemical reduction of AgNPs relies on various essential components, such as the metal precursor and a reducing agent. Additionally, the morphology and size of these NPs are influenced by both the constituents of the reaction and the manipulation of reaction parameters, such as temperature and pH.	Evanoff and Chumanov (2004), Chen and Zhang (2012), Dang et al. (2012), Iravani et al. (2014)
	Physical-based: various techniques can be employed for the physical synthesis of AgNPs, such as condensation, evaporation, and thermal degradation procedures. The ceramic heating method is employed for the purpose of generating AgNPs that possess a monodisperse and uniform size. The application of physical methods in the synthesis of AgNPs led to the attainment of AgNPs with consistent shape and size. Nevertheless, it is crucial to take into account the primary expenses associated with investing in equipment, the significant amount of time required, and the requirement for a substantial energy input.	Lee and Kang (2004), Iravani et al. (2014)
	Green approach: AgNPs are generated through the utilization of various biological entities such as bacteria, yeast, fungi, algae, and plants. These entities serve as both reducing agents and stabilizing agents. For instance, the fungus *Trichoderma viride* has been employed to synthesize AgNPs from AgNO_3_, which acts as a precursor in the process. The combination of *Fatsia japonica* leaf extract and AgNO_3_ resulted in the synthesis of AgNPs that exhibited remarkable antibacterial properties.	Sintubin et al. (2012), Iravani et al. (2014)
Iron NPs	Physical–chemical-based: there are several physical techniques available for synthesizing iron NPs, such as mechanochemical processes and vapor-based procedures. On the other hand, chemical synthesis of iron oxide can be achieved using techniques such as sol–gel synthesis, hydrothermal procedures, coprecipitation, and template-assisted synthesis. By modifying the iron salt precursor in different synthetic procedures, it is possible to synthesize various forms of iron NPs.	Carvalho et al. (2013), Ali et al. (2016)
	Green approach: the utilization of green synthesis methods for the production of iron NPs has demonstrated notable advantages in terms of simplicity, effectiveness, and reduced time requirements. An illustrative example involves the synthesis of iron NPs through the utilization of an extract derived from *Camellia sinensis* leaves. The polyphenols found in green tea extract possess qualities that enable them to effectively reduce ferric cations. Additionally, these polyphenols also serve as capping agents.	Gottimukkala et al. (2017)
Zinc oxide NPs	Chemical-based: in the chemical synthesis techniques, zinc oxide NPs can be produced using reduction processes. This involves the utilization of zinc nitrate and potassium hydroxide (KOH) in an aqueous solution, leading to the formation of NPs with a particle size ranging from 30 to 15 nm.	Ghorbani et al. (2015)
	Physical-based: To prepare this substance, physical methods such as colloidal dispersion and vapor condensation can be utilized. Another method for producing ZnO NPs involves nanosecond laser ablation of zinc. The resulting ZnO NPs typically have particle sizes ranging from 36 to 88 nm.	Aladpoosh and Montazer (2015)
	Green approach: zinc oxide NPs can be produced through a biological process that involves combining zinc acetate and sodium hydroxide with a plant extract. The process leads to the formation of spherical NPs in the size range of 23–57 nm. The biological technique offers several advantages, which include the ease of synthesis, rapid reaction time, and the absence of adverse effects.	Agarwal et al. (2017)
Copper NPs	Physical and chemical methods: these methods include vacuum vapor deposition, sonochemical reduction, pulsed laser ablation, electrochemical procedures, and hydrothermal processes.	Tamilvanan et al. (2014)
	Green approach: the biological or biosynthetic production of copper NPs has been successfully accomplished through the utilization of ascorbic acid as a dual-functioning agent, serving as both a protective and reducing agent. This approach has several advantages, which include its environmentally benign nature, cost-effectiveness, and non-toxic properties.	Umer et al. (2012)
Platinum NP	The synthesis techniques employed for the production of these NPs encompass several approaches, such as colloidal systems, reduction via formaldehyde and sodium borohydride, utilization of bacterium cellulose as a hydrophilic matrix, microemulsion, sol–gel method, sonochemical method, and electrodeposition.	Evans et al. (2003)

### 4.1 Noble metal-based nanoparticles

Noble metals refer to a group of metallic chemical elements that exhibit exceptional resistance to oxidation, even when exposed to elevated temperatures. The primary constraint associated with their application pertains to the elevated expenses associated with certain metallic elements. Gold, silver, platinum, and palladium are frequently utilized as noble metals for the creation of NPs.

**FIGURE 10:**
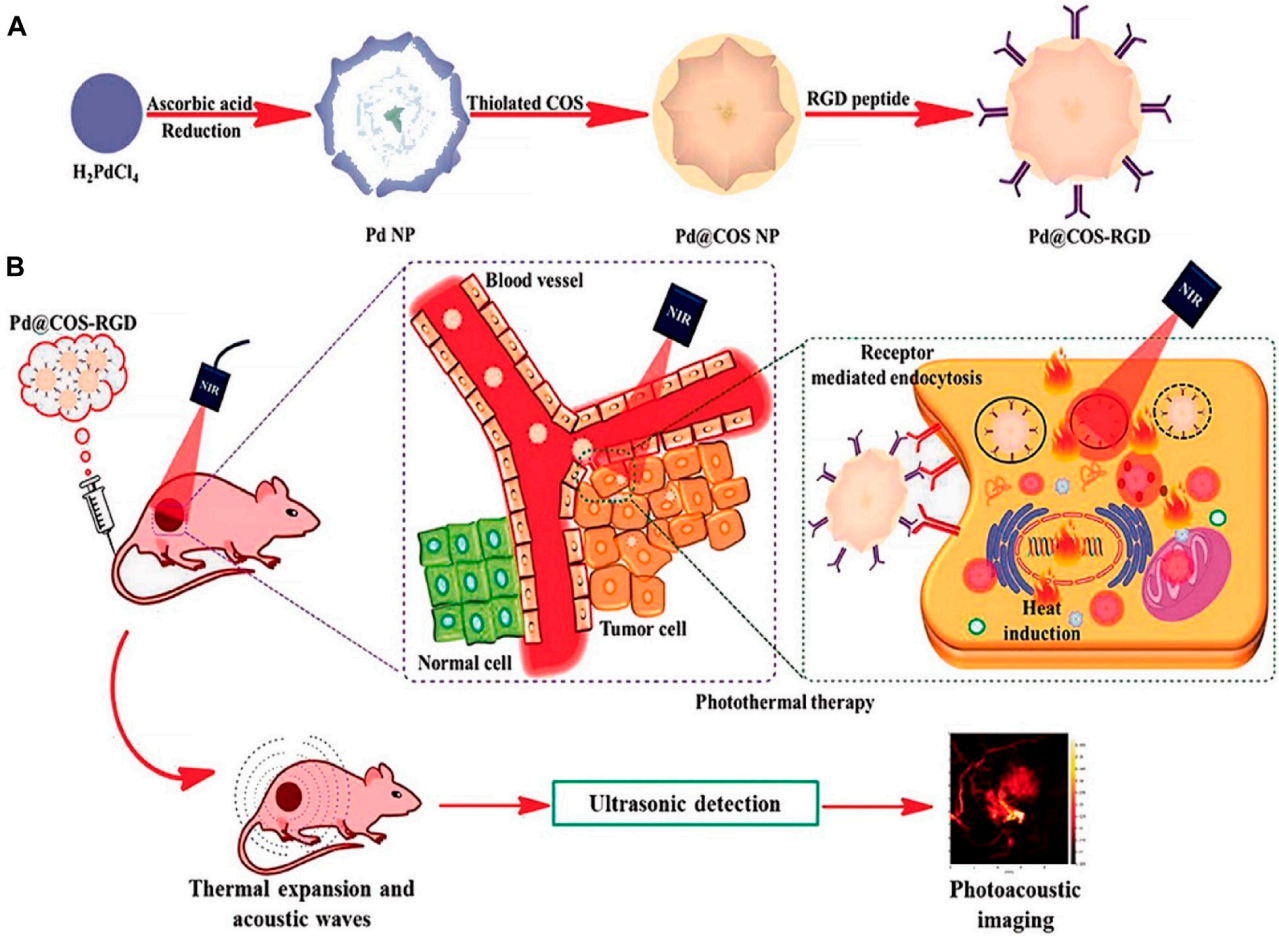
**(A)** The process involves the synthesis of Pd NPs followed by the application of a surface coating of thiolated chitosan oligosaccharide (Pd@COS NPs). Subsequently, the functionalization of the coated NPs is achieved by incorporating the RGD peptide, resulting in Pd@COS-RGD NPs. **(B)** A systematic illustration of the application of Pd@COS-RGD for photothermal ablation and photoacoustic imaging of tumor tissue (Bharathiraja et al., 2018). Reproduced with permission from Bharathiraja et al. (2018). Copyright, Nature, 2018.

- Gold nanoparticles: gold is considered a noble element due to its non-reactive properties. The material’s ability to withstand chemical oxidation makes it very resistant to degradation and corrosion. Therefore, it has the ability to maintain its physical structure and shine for thousands of years. AuNPs can be synthesized through a range of methods, which include physical, chemical, and green approaches. These techniques encompass both top–down and bottom–up approaches, which are utilized in various types of synthesis (Chen et al., 2013). The unique physicochemical features of AuNPs contribute to their extensive range of biomedical applications. In recent times, there has been a significant amount of discourse surrounding the subject of tumor targeting. By surface functionalization or coating, the anti-tumor properties of AuNPs can be enhanced. These NPs have the potential to be utilized in a range of applications such as diagnostics, therapeutics, bioimaging, and prognostics. In a study conducted by Botteon et al., the biogenesis of AuNPs was described using Brazilian red propolis, a bee product. The researchers treated T24 and PC-3 cancer cell lines with green AuNPs and observed significant cytotoxic effects *in vitro* (Bray et al., 2018). Umapathi et al. (2020) produced AuNPs with a functionalization of isonicotinic acid hydrazide corona and CUR target cancer. The researchers observed significant detrimental effects of these functional NPs, especially in LK-2 and TIG-120 cells. The anticancer activity of ROS was enhanced through the conjugation of CUR and isoniazid on AuNPs (Botteon et al., 2021).
- Silver nanoparticles: the utilization of AgNPs in the field of biomedicine has gained significant traction in recent years. The wide range of applications for AgNPs can be attributed to their diverse properties, especially as an anticancer agent (Sondi and Salopek-Sondi, 2004). The principal processes by which AgNPs operate encompass the generation of ROS, induction of oxidative stress, and infliction of DNA damage. Nevertheless, the overabundance of intracellular ROS serves as a mechanism for inducing toxicity in AgNPs since it leads to detrimental effects on DNA, lipids, and proteins (Jain et al., 2021). The toxicity observed in treated cells is due to the release of silver ions within the cytosol. This release happens when AgNPs are taken up by the cells through endocytosis and then degraded in an acidic environment. Consequently, the presence of AgNPs has been associated with an elevated susceptibility to cancer and cellular apoptosis as a result of their capacity to disrupt fundamental metabolic and cell cycle pathways within the cell. According to Muhammad et al. (2020), the utilization of AgNPs to functionalize paclitaxel nanocrystals has been found to enhance the overall efficacy of anti-cancer activity on human cancer cells. The researchers successfully synthesized nanocrystals that integrated paclitaxel with AgNPs, which were utilized as agents for targeting tumors. Polydopamine (PDA) was utilized as a coating material for the paclitaxel nanocrystals, serving as a template for their formation. The PDA layer played a pivotal role in connecting the *in situ* manufacturing and deposition of AgNPs, and in facilitating the grafting of the tumor-targeting peptide NR1, which utilized the RGDARF sequence. The utilization of drug nanocrystals coated with NR1/AgNPs has demonstrated substantial improvements in anti-cancer activity and cellular uptake. Based on the available evidence, it can be shown that the combination of AgNPs with paclitaxel exhibits an additive or synergistic effect on various aspects, such as their mutual interaction with the receptor, controlled drug release, and their tiny size. The NR1-AgNP-decorated PTX nanocrystals exhibited a favorable balance in terms of targeting and biocompatibility. The nanocrystals exhibited a high level of apoptotic efficiency, leading to the disruption of the DNA, nucleus, mitochondria, and cell membrane. The hypothesis suggests that the activation of P53 and caspase 3, along with the change in the Bax-to-Bcl-2 ratio, may play a significant role in the potential mode of action and molecular basis of these specific pharmacological nanocrystals (Muhammad et al., 2020).
- Platinum nanoparticles: platinum-based medications such as cisplatin, carboplatin, and oxaliplatin are widely employed for the treatment of patients worldwide. Nonetheless, the absence of precise targeting in cancer therapy gives rise to detrimental consequences and a rise in the development of drug resistance (Mochida et al., 2017). Platinum NPs are employed in several research investigations within the fields of biotechnology, nanomedicine, and pharmacology. The evaluation of inorganic platinum NP nanoformulations in human subjects has not yet been conducted. To potentially enhance the therapeutic efficacy of platinum NPs within the human body, it may be advantageous to employ a biocompatible coating, such as polyvinylpyrrolidone (PVP), on their surfaces (Jeyaraj et al., 2019). This is particularly relevant as the duration of circulation of these platinum NPs increases. In this research investigation, DOX was employed as a representative therapeutic compound to synthesize platinum NPs functionalized with PVP. The resulting NPs exhibited an octopod morphology and displayed a high degree of uniformity in terms of their size distribution. The utilization of the system was employed to enhance the process of drug distribution and mitigate the occurrence of toxicity. Both drug release and biocompatibility were enhanced by the platinum–DOX conjugate system (Patel et al., 2021). According to Kankala et al. (2020), it has been hypothesized that platinum NPs possess the ability to infiltrate tumors at a significant depth, hence exhibiting synergistic therapeutic benefits. This phenomenon is attributed to the catalytic activity of platinum NPs, facilitated by free radical species. Using a self-assembly process, chitosan was utilized to distribute ultrasmall platinum NPs onto zinc-doped mesoporous silica NPs. The incorporation of zinc species into the siliceous frameworks facilitated a more efficient loading of DOX molecules inside the acidic milieu of the tumor, eliminating the necessity for supplementary functionalization. The efficacy of anticancer treatment was enhanced through the disruption of established coordinating relationships between the host and guest species. The utilization of this approach significantly enhanced the eradication of tumors by enabling effective infiltration into deep tumor regions, while concurrently inducing the production of harmful free radicals to eliminate multidrug-resistant malignancies (Kankala et al., 2020).
- Palladium nanoparticles: palladium NPs possess remarkable catalytic and optical properties, rendering them suitable for theragnostic purposes. According to researchers, the utilization of palladium nanomaterial has been observed in many applications such as prodrug activation, photothermal therapy, as well as in anticancer and antibacterial treatments. Figure 10 showcases the utilization of palladium NPs with multifunctional capabilities, namely, in the context of photothermal treatment and imaging (Bharathiraja et al., 2018). There is a claim that palladium NPs may be manufactured by a cost-effective process using Saudi propolis. Palladium NPs demonstrated a significant therapeutic effect on MCF-7 ductal carcinoma, as evidenced by their IC_50_ value of 104.79 μg/mL (Al-Fakeh et al., 2021). The palladium NPs have been subjected to modification for the purpose of treating MCF7 breast cancer cells. This modification involves the utilization of PVP-functionalized palladium. The viability of breast cancer cells was significantly reduced with increasing doses of PVP-palladium NPs. The hypothesis suggests that the system induces cell death by mediating caspase 3/7 enzymatic activity, resulting in the impairment of DNA and mitochondrial events (Ramalingam et al., 2020).

### 4.2 Non-noble metal-based nanoparticles

Despite their inherent susceptibility to oxidation, non-noble metals offer several advantages such as affordability, abundance, and high conductivity. The following section will explore the application of these metallic elements in the field of cancer therapies.

- Magnetic nanoparticles (iron/nickel): feasible manipulation of magnetic NPs is achieved by applying external magnetic fields. These particles primarily consist of magnetic materials like iron or nickel, and functional compounds (Edis et al., 2021; Souri et al., 2021). By generating heat from the NPs, the application of a high-frequency magnetic field has the potential to increase the temperature of the tumor to a range of 40°C–46°C. One notable potential of magnetic NPs lies in their capacity to synergistically integrate hyperthermia and medication release for the purpose of cancer treatment (Dwivedi et al., 2020). The study employed magnetic NPs that consisted of DOX–gelatin cores and Fe_3_O_4_–alginate shells for the purpose of delivering specific anticancer medications. To achieve a high level of encapsulation efficiency, DOX was used as a representative drug and introduced into the gelatin core. The attainment of controlled drug release was facilitated with the utilization of an external magnetic film, hence enabling tumor targeting. The aforementioned NPs were detected within the MCF-7 cancer cells under the influence of an externally applied magnetic field. The MCF-7 cancer cells were effectively targeted by the NPs with the application of an external magnetic field. Following a 6-h incubation period, the NPs were shown to localize into the nuclei of the aforementioned cells. The viability of MCF-7 cells decreased to 52.3% following a 12-h treatment, accompanied with a relative fluorescence intensity of 98.4% (Huang C.-H. et al., 2020). The process involved the conjugation of the anticancer medication telmisartan (TEL) with Fe_3_O_4_ magnetic NPs by the use of chitosan, a biodegradable and hydrophilic polymer found naturally. The drug-loaded magnetic NP–chitosan composite (MNP-CS) demonstrated controlled release capabilities that were responsive to changes in pH. Statistically significant observations were made regarding the role of dose of MNP-CS-TEL for treating PC-3 cancer cells, highlighting the potential anticancer properties of the nano-formulation (Dhavale et al., 2021). The incorporation of magnetic NPs into self-assembled hybrid NPs offers two notable benefits: the ability to accommodate significant quantities of pharmaceuticals and the power to regulate drug release. In the present scenario, nickel ferrite (NFO) NPs have been employed for the purpose of administering anti-cancer medications. Joshy et al. (2020) demonstrated the intracellular transport of zidovudine (AZT) using NFO-reinforced hybrid NPs. To achieve this, a hybrid material composed of polyvinyl alcohol and stearic acid, incorporating NFO-containing polyethylene glycol, was utilized for the distribution of AZT. Furthermore, there is a strong focus on the utilization of green chemistry principles in the production of magnetic NPs. A study was conducted on the manufacture of nickel oxide NPs using Arabic gum as a green and sustainable method. The cytotoxic effects of nickel oxide NPs on U87MG cancer cell lines were evaluated using the MTT technique. The resulting IC_50_ value for U87MG cancer cells was determined to be 37.84 g/mL (Sabouri et al., 2021).
- Zinc oxide nanoparticles: zinc oxide is widely recognized as one of the most prevalent metallic NPs globally. Zinc oxide NPs have garnered significant interest in recent times due to their capacity to generate ROS upon light exposure. Various chemical strategies can be employed to enhance the photocatalytic effectiveness and ROS generation capabilities of zinc oxide particles. The enhanced antibacterial and anticancer properties of modified zinc oxide NPs can be attributed to their increased effectiveness in generating ROS. These modifications are achieved through techniques such as metal doping and the use of organic photosensitizing compounds (Sivakumar et al., 2018). The objective of a study was to assess the anticancer activity of zinc oxide NPs incorporating with CUR on the RD cell line. The MTT assay was employed for this evaluation. The cytotoxic effects of these NPs were also evaluated on embryonic kidney cells using the resazurin assay. The enhanced cytotoxicity of the NPs was attributed to the significant aspect ratio exhibited by the ZnO structures (Perera et al., 2020). Another study showed the utilization of egg albumin in the manufacturing process of zinc oxide NPs. The MTT assay was used to evaluate the anticancer activity of the system on MCF-7 cells. The results indicated significant cytotoxicity and a proportional decrease in cellular viability. According to gene expression research utilizing Western blot and RT-PCR analysis, the administered NPs stimulated the production of ROS. As a result, the treatment led to an increase in the mRNA-level transcription, such as caspase-3 and -9, bcl-2, and p53. Simultaneously, there was a significant downregulation in the expression of the anti-apoptotic gene Bcl-2. The results indicated that the nano system exhibited targeted inhibition of MCF-7 gene expression by the induction of cytotoxicity, cell death, and oxidative damage mediated by ROS (Vijayakumar et al., 2020).
- Copper nanoparticles: copper is an essential element in the metabolic processes of both plants and animals. In its natural state, the substance possesses inherent qualities of softness, malleability, and pliability, alongside notable thermal and electrical conductivity properties. In comparison to similar metals, copper NPs exhibit a relatively lower cost within the group of transition metals being examined (Reddy, 2017). There has been a growing emphasis on the green synthesis of copper NPs in recent years. The utilization of broccoli green extract has been characterized as a sustainable and ecologically sound precursor for the one-pot manufacture of copper NPs. The efficacy of the formulated medication in the management of prostate cancer has been demonstrated (Reddy, 2017). A study aimed to evaluate the cytotoxic effects of a nanocomposite based on copper, silver, and chitin on MCF-7 cells. The observed inhibitory concentration of the system was determined to be 31 mg. Additional research findings demonstrated an elevation in ROS generation, a reduction in antioxidant enzyme functionality, and a deterioration of membrane integrity, thereby corroborating the detrimental impact on cellular function caused by the nanocomposite composed of copper NPs and AgNPs (Solairaj et al., 2017).
- Cerium oxide nanoparticles: cerium oxide NPs encapsulated within an oxygen lattice have demonstrated promising capabilities across a diverse range of applications. These substances have the ability to trigger apoptosis in cancer cells (Gao et al., 2014). Cerium oxide NPs have been found to enhance the sensitivity of pancreatic cancer cells to radiation therapy by activating the JNK apoptotic pathway through oxidative mechanisms. Cerium oxide NPs have been observed to induce an upregulation of ROS production in cancer cells. The initiation of apoptosis signaling kinase 1 (ASK1) activation was demonstrated to be triggered by ROS, which oxidize thioredoxin 1 (TRX1). It was hypothesized that the activation of JNK is augmented due to the observed rise in TRX1 oxidation, as supported by the increased activation of ASK1 after co-treatment with cerium oxide NPs and RT (Wason et al., 2018).
- Titanium nanoparticles: titanium dioxide exists in nature in several forms, namely, anatase, brookite, and rutile, which are considered inert minerals. These minerals are found in different quantities. Numerous methodologies exist for the synthesis of titanium dioxide NPs, encompassing both physical and chemical techniques. The methods employed for the production of nanomaterials exhibit some limitations, such as high costs, limited biocompatibility, various secondary toxicities, and significant environmental biosafety concerns. The concept of biogenesis has been proposed in the synthesis of natural products. In the production of titanium dioxide NPs, various species of plant materials are utilized for biogenesis. Biogenic titanium dioxide NPs exhibit unique characteristics in physicochemical and biological functional properties. These attributes enable them to exert therapeutic effects at the molecular level, which include anticancer activities (Ikram et al., 2021). Mohammad et al. (Behnam et al., 2018) worked on melanoma cancer therapy using the application of titanium dioxide NPs in photothermal therapy. In the experimental animal model, a notable reduction in the mean tumor size was observed in mice treated with titanium dioxide–PEG NPs combined with laser stimulation, as compared to animals subjected to laser therapy alone.

## 5 Pharmacokinetics and biodistribution of metal-based nanoparticles

In order to fully harness the extensive potential and therapeutic applicability of metallic NPs, it is imperative to conduct a comprehensive evaluation of their overall impact prior to their implementation in clinical settings. Further investigations are required to assess the PKs and biodistribution of nanomaterials in order to gain a comprehensive understanding of their tolerance range and potential adverse reactions. The absorption and behavior of these nanomaterials in biological systems can be further modified by their size, shape, and ligand composition. Therefore, this section analyzes the consequences of nanoscale materials on biological systems and the subsequent impact on the surrounding environment.

### 5.1 Mode of administration

A comprehensive comprehension of the interactions between NPs and biological systems *in vivo* is imperative for the successful utilization of NPs as medicinal agents. Accurate characterization of nanomaterials requires the utilization of an appropriate *in vivo* model and rigorous statistical analysis. The route of administration plays a significant role in the variations observed in the PKs and toxicity profile of NPs. Several studies have provided evidence indicating that the absorption of gold through intramuscular and intravenous injections is comparatively higher than oral administration (Gottlieb, 1983; Champion et al., 1990). The toxicity and PK profiles of auranofin and gold sodium thiomalate have been extensively reported. However, there is still an ongoing controversy regarding the optimal mode of administration (Kean and Kean, 2008). The maximum absorption rate of injected gold was observed to be only 20%–25% after a 2-h period. However, administering gold intermittently resulted in inconsistent concentration levels among individuals (Blocka et al., 1982; Walz et al., 1983). The administration of gold complexes through intravenous injection has been observed to result in dermal buildup and corneal deposition, in contrast to the oral route of administration (Penneys et al., 1975). On the other hand, gold complexes delivered orally have a prolonged half-life and maintain a consistent concentration in the blood plasma throughout the duration of the treatment. Moreover, a significant proportion (approximately 85%–95%) of the orally supplied gold complex is eliminated through excretion. Nevertheless, the residual quantity (5%–15%) is excreted via the urinary system (Gottlieb et al., 1972). Brandau group examined the biodistribution of AuNPs with diameters of 1.4 nm and 18 nm using two different modes of administration: direct and intravasation delivery (Semmler‐Behnke et al., 2008). Upon analysis of the results, it was discovered that the smaller NP showed translocation along the respiratory tract after being delivered directly, whereas the 18-nm particle remained localized in the lungs. Following intravenous injection, it was shown that both NPs exhibited accumulation in the liver. Notably, the 18-nm AuNPs demonstrated a twofold increase in accumulation compared to the other NPs, with a value of 93.6% injection dosage (ID)/g (Semmler‐Behnke et al., 2008). The research conducted by the Zhang et al. (2010) revealed that the toxicological characteristics of 13.5 nm AuNPs are influenced by the method of delivery. Mice were administered a dosage range of 137.5–2,200 μg/kg by oral, intraperitoneal (IP), or tail vein injection routes. The administration of injections through the tail vein resulted in negligible harmful effects, as evidenced by small changes observed in the counts of white blood cells and platelets. Furthermore, the alteration in hemoglobin concentration did not yield a statistically significant outcome. Nonetheless, it was observed that the administration of substances intraperitoneally and orally resulted in heightened toxicity, accompanied by a decrease in the count of red blood cells (Zhang et al., 2010).

In a study, the biodistribution of orally delivered silver salts and silver acetate NPs in rats was compared (Loeschner et al., 2011). Despite differences in formulation, the distribution of silver salts and silver acetate in organs showed similarities. The excretion of silver resulting from the treatment with AgNPs was found to be much higher in fecal matter (63%) than in urine (0.005%). The concentration of silver in biliary fluid was found to be 16 to 20 times higher than was in rat plasma. The application of Authometalliographic (AMG) staining revealed the presence of silver exclusively on the external layer of intestinal villi located in the ileum, whereas no silver was observed within the cellular cytoplasm. There was a significant AMG grain staining observed in both the glomeruli and renal tubules of the renal papilla. Notably, there was no noticeable difference in the staining pattern between mice exposed to silver acetate or AgNPs (Loeschner et al., 2011). The biodistribution of polyvinylpyrrolidone-coated and radioiodine-labeled AgNPs with a diameter of 12 nm was investigated in BALB/C mice through intravenous injection, as described by Chrastina and Schnitzer (2010). The Computed Tomography - Single Photon Emission Computed Tomography (CT-SPECT) imaging technique demonstrated that the majority of particles were absorbed by the reticuloendothelial system, with 41.5% accumulating in the spleen and 24.5% in the liver, within a 24-h timeframe. The remaining particles were found to be sparsely dispersed among the other organs. The particles observed demonstrate a migration process from the initial injection site, followed by dispersion to a secondary location (Chrastina and Schnitzer, 2010). Nevertheless, alternative sources suggest an augmentation in liver enzyme activity, heightened absorption by resident macrophages, escalated inflammatory reaction, and hepatic impairment. The comprehensive investigation clearly illustrated that the toxicity of nanosilver is dependent on the method of delivery. Furthermore, it should be noted that the toxicological impacts of AgNPs are contingent upon both the dosage administered and the duration of exposure (Chrastina and Schnitzer, 2010).

The potential impact of NP inhalation on biological systems warrants a quick examination. The inhalation-induced toxicity of AgNPs was documented in the animal experiments conducted by Sung et al. (2008, 2009, 2011) employing Sprague–Dawley rats as the animal model (Sung et al., 2008; Sung et al., 2009; Sung et al., 2011). The earliest investigations conducted by Sung et al. (Sung et al., 2008) showed the potential biological effects of prolonged exposure to 18 nm AgNPs. Female and male rats were exposed to these NPs for a 90-day period. The investigation’s findings demonstrated that the use of AgNPs results in a decrease in pulmonary function and the formation of inflammatory lesions in the lungs. Notably, these effects were observed at much lower mass dose concentrations (2.9 × 106 particles/cm^3^) than submicrometer particles. In the subsequent investigation, it was revealed that there was a dose-dependent rise in bile duct hyperplasia in the liver of both male and female rats, when subjected to similar experimental settings (Sung et al., 2009). In the latest study, rats were subjected to a 4-h exposure of 18-nm AgNPs within a whole-body inhalation chamber, followed by a subsequent observation period of 2 weeks. After conducting a thorough evaluation of lung function, the study found no evidence about acute toxicity because of the inhalation of AgNPs (Sung et al., 2011).

### 5.2 Particle size and shape

To ensure the safety and effectiveness of nanoformulation, it is imperative to consider various physiochemical factors of NPs and the characteristics of the binding ligand. This careful consideration is crucial for minimizing potential toxicity and maximizing the therapeutic index. The initial study conducted by Hillyer demonstrated an inverse relationship between the size of orally administered AuNPs and their dispersion inside the body. Geertsma’s study revealed that AuNPs measuring 10 nm were widely distributed throughout the body, while larger NPs were specifically found in the circulation, liver, and spleen (De Jong et al., 2008). Pan et al. conducted a separate investigation and found that NPs with a diameter of 1–2 nm exhibited significant toxicity across several cell types. However, they observed that 15-nm gold particles had comparatively minimal toxicity (Pan et al., 2007). Additionally, the scientists demonstrated that the cellular response exhibits a dependence on particle size, even when the particles are of comparable size. In a study conducted by Abdelhalim and Jarrar (2011), it was shown that the administration of AuNPs with sizes of 10, 20, and 50 nm to Wistar–Kyoto rats resulted in hepatotoxicity and renal toxicity. The rats were subjected to a dosimetry period of either 3 or 7 days. The harmful effect of smaller particles was shown to be greater than that of bigger particles. This was attributed to the formation of ROS, which resulted in necrosis, changes in renal tubules, increased hyperplasia of Kupffer cells, and rupture of the intima of central veins. A pioneering investigation conducted by Chen et al. (2009) examined the impact of uncoated NPs of sizes of 3–100 nm administered intraperitoneally (IP260). The BALB/C mice were administered with a dosage of variant AuNPs at a rate of 8 mg/kg each week. The study revealed that particles ranging from 8 to 37 nm in size induced acute toxicity in mice. This toxicity was characterized by several symptoms such as fur color change, crooked spine, lack of appetite, and reduced body weight after a period of 14 days. These effects were observed in contrast to the normal mice employed as the control group. Nevertheless, the observed toxicity levels were not visible for particle core sizes of 3, 5, 50, and 100 nm. The histopathological investigation revealed an augmented presence of Kupffer cells within the hepatocytes, and structural degradation observed in the lungs, spleen, and liver. By utilizing AuNP enhancement, the presence of AuNPs at these specific locations was confirmed through the detection of an amplified Coherent anti-Stokes Raman signal. These findings provide evidence for the aforementioned observations (Chen et al., 2009).

Park et al. (2010) investigated the toxicity of AgNPs in a size-dependent manner. The researchers exposed mice to varying sizes of AgNPs (22, 42, 71, and 323 nm) at a dose of 1 mg/kg through oral administration for a duration of 2 weeks. The particles with a diameter of 22 nm exhibited enhanced toxicity, as seen by greater infiltration of immune cells (specifically B cells and CD8^+^ T cells) and elevated levels of TGF-β and cytokine release. By contrast, the particles with bigger sizes did not elicit any detrimental effects (Park et al., 2010). Organ weight remained unchanged in all the treatments, regardless of the size, as compared to the control group. The number of 22-nm particles detected in the brain tissue, which was potentially capable of crossing the blood–brain barrier, was found to be greater than that of bigger particles, which exhibited limited distribution inside the brain. Additionally, it was shown that the toxicity of 42-nm particles exhibited a dose-dependent relationship with doses of 0.25 mg/kg, 0.5 mg/kg, and 1.0 mg/kg. Notably, the highest treatment dosage resulted in elevated levels of alkaline phosphatase, aspartate transaminase, and alanine transaminase. The histopathological examination revealed slight cortical injury in the kidneys, whereas no significant alterations in the morphology were observed in the liver and small intestines (Park et al., 2010). Lankveld et al. (2010) conducted a study, to investigate the biodistribution of AgNPs. The authors administered the NPs intravenously for 5 consecutive days and presented their findings. The study also included three different sizes of NPs (20, 80, and 110 nm). The biodistribution was studied for a total of 16 days. The NPs exhibited prompt clearance from the bloodstream and distribution across several organs, regardless of their sizes. The larger particles exhibited a higher tendency to concentrate in the spleen, with subsequent accumulation observed in the liver and lungs. Conversely, the smaller particles (20 nm) demonstrated a primary deposition in the liver, followed by subsequent distribution to the kidneys and spleen (Lankveld et al., 2010).

### 5.3 Effect of surface chemistry

The impact of NPs’ surface chemistry on PKs and biodistribution is very significant. This information has the potential to offer design concepts that can be employed to enhance the delivery of therapeutic agents specifically to tumors. In a particular study, mice were subjected to injections of AuNPs of varying sizes (ranging from 5 to 22 nm) (Balogh et al., 2007). These NPs were categorized into five categories and possessed surface charges that were either positive, negative, or neutral. After conducting an examination of blood, excrement, and various tissues, it was found that the concentration of positively charged 5-nm AuNPs was higher in the blood following injection than other charged NPs. Furthermore, it was revealed that the positive NPs exhibited the highest concentration in the kidneys, with a value of 24% ID/g, indicating that it was effectively confined in this organ (Balogh et al., 2007). Statistically, considerable buildup of negatively and neutrally charged particles is observed in the liver in comparison to other organs. Biodistribution investigations conducted on several strains of mice, which included immunodeficient and immunocompetent strains, have revealed that the surface charge of AuNPs and the methods by which they are systemically administered have distinct effects on their PKs, distribution throughout organs, and uptake by tumors (Arvizo et al., 2011b). Neutral and zwitterionic particles exhibit a favorable PK profile characterized by high systemic exposure and limited clearance upon intravenous injection. The NPs that were delivered intraperitoneally exhibited significantly reduced systemic exposure compared to the NPs that were administered intravenously. The inability of the particles to cross the peritoneal barrier suggests that they cannot traverse this barrier. In a subcutaneously implanted xenograft model of ovarian cancer, the neutral and zwitterionic NPs exhibited increased tumor uptake. This enhanced uptake can be attributed to the low plasma clearance observed for both treatment routes. In addition, intravenous administration of AuNPs resulted in significant accumulation primarily in the liver, with subsequent accumulation observed in the spleen and kidneys. Notably, AuNPs with positive charge exhibited the lowest accumulation. Conversely, IP administration of AuNPs led to notable concentration in the pancreas, followed by the reticuloendothelial system (Arvizo et al., 2011b). The biodistribution of particles can be influenced by both their particle size and surface charge. Hirn et al. (2011) conducted a study wherein rats were administered intravenous injections of AuNPs that were labeled with radioisotopes. The NPs in this study had a range of sizes, from 1.4 to 200 nm, and were distinguished by their positive or negative charges. The biodistribution of the negatively charged AuNPs was found to be dependent on their size, with the highest concentration observed in the liver. On the other hand, the positively charged particles exhibited a more varied distribution pattern. Furthermore, a hypothesis was proposed suggesting that the uptake of the NPs could be influenced by the process of protein binding and exchange occurring on their surface (Hirn et al., 2011). In a study conducted by Zhu et al. (2010), zebra fish were subjected to exposure of AuNPs with a diameter of around 10 nm. The NPs in question had different surface charges, such as hydrophilic neutral, negative, positive, and hydrophobic positive charges. The exposure duration varied across three time intervals: 24, 48, and 72 h. Over the course of the study, it was demonstrated that the uptake of positive NPs was more pronounced than the uptake of negative and neutral AuNPs. Nevertheless, the fish that were subjected to hydrophobic AuNPs experienced mortality within a span of 24 h (Zhu et al., 2010). The AuNPs with positive, negative, and neutral charges exhibited a predominant accumulation in the intestinal region. Furthermore, the positively charged AuNPs were eliminated through excretion, whereas the neutrally charged AuNPs exhibited a tendency to remain within the body. On the contrary, the hydrophobic AuNPs displayed a broader distribution, with the gills, heart, and dorsal fin exhibiting the highest concentration. These results emphasize the strategic approach taken in the development of these NPs, which aimed to mitigate the toxicity commonly associated with hydrophilic NPs (Zhu et al., 2010).

## 6 Clinical translation of metal-based nanoparticle

Despite the preclinical advancements discussed above, the FDA approval process poses substantial challenges for the utilization of metallic NPs in cancer therapeutics, which have not been overcome thus far (Anchordoquy et al., 2017). Comprehensive guidance about the translation of metallic NPs has not been provided by the FDA due to the limited number of candidates that have undergone clinical evaluation for medicinal purposes (Table 8). The regulation of NPs necessitates the comprehensive assessment of each constituent’s safety, leading to more costly studies than those conducted for conventional small-molecule therapies. The Nanotechnology Characterization Laboratory facilitates partnerships between investigators and the FDA with the objective of minimizing obstacles to clinical progress for organizations involved in these trials. Additionally, accepted applicants are provided with preclinical toxicological evaluations without any financial burden on the investigator. However, due to the financial burden involved in developing these formulations and the lack of an established precedent for approving metallic NPs, researchers have been discouraged from actively pursuing clinical translation (Metselaar and Lammers, 2020; Souri et al., 2022b). Despite the desire of investigators to further the clinical translation of metallic NPs, there exist limited financing sources and research incentives for such endeavors. Despite extensive study spanning several decades and substantial financial investment from the federal government, significant initial approval from the FDA for a therapy utilizing metallic NPs has not been attained. With the current advancements, it has become more difficult to justify the prioritization of metallic NP therapies over biodegradable NP delivery systems like polymeric or liposomal approaches. Indeed, a considerable number of notable organizations that prioritize clinical translation have transitioned toward employing non-metallic particles in the development of translational medicines (Anselmo and Mitragotri, 2015; Weissig and Guzman-Villanueva, 2015; Souri et al., 2024d).

**TABLE 8:** Metal NPs in cancer treatment in clinical trials and FDA approval, which include formulations, efficacy, application, and status.

Metal NPs	Formulation	Efficacy	Application	Status	Ref.
Gold NPs	Aurimmune	Enhanced targeting and uptake by cancer cells and reduced tumor growth	Breast cancer	Clinical trials (Phase I/II)	Alphandéry et al. (2015)
AgNPs	SilGen	Induces apoptosis and inhibits cancer cell proliferation	Lung cancer	Preclinical studies	Sahoo et al. (2023)
Iron oxide NPs	FERAHEME	Improved imaging contrast and targeted drug delivery	Multiple cancer types	FDA approved (for imaging)	Zhu et al. (2017)
Platinum NPs	Platin-M	High cytotoxicity to cancer cells and reduces tumor size	Ovarian cancer	Clinical trials (Phase II)	Duan et al. (2016b)
Silica NPs	AuroShell	Heat generation under infrared light and selective destruction of cancer cells	Head and neck cancer	Clinical trials (Phase I)	Amendoeira et al. (2020)
Zinc oxide NPs	ZnO-NP	Induces oxidant stress in cancer cells and enhances radiotherapy effects	Prostate cancer	Preclinical studies	Anjum et al. (2021)
Cerium oxide NPs	CNP-TumorX	Anti-oxidant properties and reduces inflammation and tumor growth	Colorectal cancer	Clinical trials (Phase I)	Datta et al. (2020)
Titanium dioxide NPs	TiO_2_-NanoGel	Photodynamic therapy and generates ROS	Skin cancer	Clinical trials (Phase I)	Atta et al. (2017)
Gadolinium NPs	Gd-NP	Enhanced MRI contrast and targeted drug delivery	Brain cancer	Clinical trials (Phase I/II)	Garcia-Prada et al. (2023)

The translatability of metallic NP therapeutics has raised concerns due to recent evidence highlighting their long-term *in vivo* biocompatibility issues (Table 9). These concerns have been further compounded by the presence of side effects and the lack of progress observed in clinical trials. One specific adverse effect that has been documented is the manifestation of argyria in individuals who have been exposed to colloidal silver. It is important to restructure and reevaluate the use of metallic NP therapeutics to address these concerns and ensure safer and more effective treatments (Blumberg and Carey, 1934). The examination presents a case study of a patient who developed hyperlipidemia, diabetes, hypertension, and a blue-gray facial coloration following the ingestion of colloidal silver on a triannual basis over a period of 2 years. A recent report has surfaced detailing more cases of neurological disorders in a 75-year-old male subject who practiced self-administration of colloidal silver for medicinal purposes (Tang and Xi, 2008). The administration of AgNPs through inhalation over a period of 14 days has the capacity to stimulate the increased expression of 468 genes in the cerebrum. Within this set, several genes were shown to be linked to several neurodegenerative disorders (Lee et al., 2010). Furthermore, it has been noted that the enzymes found in saliva possess the capability to convert gold (0) to gold (I), leading to its subsequent absorption by immune cells (Rapson, 1985). Typically, the observed toxicity linked to gold (I) compounds mostly presents as cutaneous and mucosal hypersensitivity, characterized by the emergence of macular and papular rash, eosinophilia, erythema nodosum, and several other manifestations of allergic responses (Merchant, 1998). The occurrence of diarrhea has been observed as a potential adverse reaction associated with the oral administration of gold complexes in specific cases. There have been recorded instances of nephrotoxicity occurring minimally in cases when modest proteinuria has been observed subsequent to the administration of gold complexes. Gold complexes possess the capacity to elicit hematological complications and are contraindicated for pregnant individuals owing to their teratogenic attributes. There have been findings suggesting that the systemic transportation of NPs can potentially lead to the induction of thrombosis, hemolysis, and other immunogenic reactions (Dobrovolskaia and McNeil, 2007).

**TABLE 9:** A summary of toxicity issues related to various metal NPs.

Metal NP	Toxicity concerns	Mechanisms of toxicity	Factors influencing toxicity	Mitigation strategies
AuNPs	Generally low toxicity but can cause oxidative stress and inflammation at high concentrations	- Induction of oxidative stress - Inflammatory responses - Cellular uptake and accumulation	- Size and shape of NPs - Surface chemistry and coating - Dosage and exposure duration	- Surface modification with biocompatible polymers (e.g., PEGylation) - Controlled dosage
AgNPs	High toxicity; induces cytotoxicity, genotoxicity, and apoptosis	- Release of Ag + ions - Oxidant stress - Interaction with cellular components	- Particle size - Ag + ion release rate - Surface coating and functionalization	- Use of stabilizing agents to control ion release - Employing lower and effective doses
Iron oxide (Fe_3_O_4_) NPs	Generally considered safe but can cause oxidative stress and inflammatory responses at high doses	- Generation of ROS - Disruption of cellular functions	- Size and surface properties - Coating material (e.g., dextran and silica) - Aggregation state	- Coating with biocompatible materials - Optimization of particle size and dose
Zinc oxide (ZnO) NPs	High toxicity; induces oxidative stress, cytotoxicity, and genotoxicity	- Dissolution and release of Zn^2+^ ions - ROS generation - Membrane damage	- Particle size and shape - Zn^2+^ ion release - Surface modifications	- Surface modification to reduce ion release - Use of antioxidants to mitigate ROS
Copper (Cu) NPs	High toxicity; causes oxidative stress, cytotoxicity, and inflammatory responses	- Release of Cu^2+^ ions - ROS generation - Interaction with cellular components	- Size and surface area - Cu^2+^ ion release - Surface coating	- Surface modification to control ion release - Application of lower, effective doses
Platinum (Pt) NPs	Moderate toxicity; can cause oxidative stress and cellular toxicity at high doses	- Interaction with cellular components - ROS generation - DNA damage	- Particle size and surface characteristics - Dosage and exposure duration	- Surface coating with biocompatible materials - Dosage control
Titanium dioxide (TiO_2_) NPs	Low-to-moderate toxicity; potential to cause oxidative stress and inflammation	- ROS generation under UV exposure - Cellular uptake and accumulation	- Crystal structure and size - Surface coating and functionalization - Exposure to UV light	- Use of UV-blocking agents - Surface modification to enhance biocompatibility
Gadolinium (Gd) NPs	Potential for nephrotoxicity and fibrosis, particularly in patients with renal impairment	- Release of Gd^3+^ ions - Interaction with cellular components - Induction of fibrosis	- Gd^3+^ ion release - Surface coating - Patient’s renal function	- Use of stable chelating agents - Monitoring renal function before administration

Given the challenges associated with the clinical translation of metallic NPs, it is imperative for researchers seeking to have a therapeutic impact to provide compelling rationale for utilizing metallic NPs over polymeric and liposomal formulations. Metallic NPs have distinct advantages in several scenarios, such as therapeutic interventions that exploit the optical characteristics of metallic NPs for ablation purposes, or the utilization of the inherent immune-stimulating features of metallic NPs in the context of cancer immunotherapy applications. The investigation of the interactions between NPs and the immune system has garnered increased attention in light of the recent advancements in cancer immunotherapy (Dobrovolskaia et al., 2016; Dykman and Khlebtsov, 2017). Initial findings indicate that NPs have the potential to induce both humoral and cellular immune responses independently, without the requirement for additional immune stimulants. Consequently, it is imperative to conduct more investigations to better understand the mechanisms via which NPs initiate immunological stimulation (Lee et al., 2014). Additional research is necessary to enhance the efficacy of NPs in the context of immunotherapeutic applications by gaining a more comprehensive understanding of the interactions between metallic NPs and immunological microenvironment.

## 7 Conclusion

Metal NPs have been the subject of substantial research owing to their exceptional and versatile physical and chemical characteristics. The present review has resulted in the attainment of lessons learned and challenges associated with metal NPs, such as their cost-effectiveness, simplicity of synthesis, and the ability to manipulate their shape and dimensions, and clinical translation (Table 10). Additionally, noble metal NPs possess unique plasmonic characteristics that make them a reliable tool for monitoring therapeutic carriers at the nano level within the human body. This capability enhances the effectiveness of therapy and reduces the risk of adverse effects compared to traditional treatment methods. The use of these NPs allows practitioners to diagnose and track treatment progress more accurately throughout the entire process. On the other hand, non-noble metal NPs offer cost-effective alternatives and exhibit distinct features such as hyperthermia and magnetic properties. By restructuring the approach, the potential of both noble and non-noble metal NPs can be harnessed to advance therapeutic interventions. Numerous research studies have provided evidence on the effectiveness of metal NPs as a promising avenue for cancer treatment. Presently, several compositions of metal NPs are undergoing preclinical and clinical trials. However, there are still numerous challenges that require attention and resolution. The tumor imaging procedure will employ metallic NPs to accurately ascertain the specific stage of the tumor. Additionally, efforts will be made to devise tumor therapy techniques that eliminate the toxicity levels commonly associated with current methodologies. Nevertheless, it is imperative to evaluate multiple factors pertaining to the production and utilization of these substances prior to their implementation in clinical studies. The precautions encompass several aspects such as the regulation of preparation methods, ensuring consistency, stability, appropriate dosage, desired accumulation at the intended site, and minimizing off-target effects. Of utmost importance is the consideration of toxicological dangers. The involvement of regulatory bodies is crucial in the development of novel criteria for the clinical application of metal NPs in cancer therapy and medication delivery, as well as in the implementation of innovative methods to assess the efficacy and safety of these NPs. Metal NPs, however, are expected to emerge as a significant clinical instrument in the battle against cancer once these issues have been addressed.

**TABLE 10:** Summarizing the lessons learned and challenges in various aspects of using metallic NPs in cancer therapy.

Aspect	Lessons learned	Challenges
Targeting and delivery	- Functionalization with ligands and antibodies can improve targeting specificity.	- Avoiding off-target effects and ensuring efficient delivery to tumor sites.
Treatment efficiency	- Metallic NPs show high efficacy in targeting and killing cancer cells via hyperthermia and drug delivery.	- Achieving consistent and reproducible results across different studies and patient populations.
Drug loading capacity	- High drug-loading capacity allows for controlled and sustained drug release.	- Ensuring uniform drug-loading and release profiles in complex biological environments.
Synthesis	- Advanced synthesis techniques allow for the production of uniform and functional NPs.	- Scalability and reproducibility of synthesis processes for large-scale production.
Physicochemical	- Precise control over size, shape, and surface properties can enhance targeting and reduce side effects.	- Maintaining stability and preventing aggregation under physiological conditions.
Safety	- Surface modification can reduce cytotoxicity and improve biocompatibility.	- Potential for long-term toxicity and accumulation in the body if not properly designed and monitored.
Biocompatibility	- Engineering NPs with biocompatible coatings improves their interaction with biological systems.	- Individual variations in patient responses and immune reactions to NPs.
Cost	- Cost-effective synthesis methods can make metallic NPs more accessible for clinical use.	- High costs associated with the production and quality control of metallic NPs.
Clinical translation	- Several preclinical studies show promising results, with some NPs entering clinical trials.	- Bridging the gap between laboratory findings and clinical application due to complex regulatory requirements.
Regulatory approval	- Understanding and addressing regulatory requirements is essential for clinical approval.	- Extensive testing required to meet safety and efficacy standards set by regulatory bodies.

Future directions for the development of metal NPs in cancer treatment should prioritize enhancing specificity and reducing toxicity through advanced surface chemistry and biocompatible materials. The design of ligands that target specific cancer cell receptors will enable precise delivery of therapeutic agents, while biodegradable materials and careful consideration of NP size will minimize side effects and ensure safe clearance from the body. Emphasis should also be placed on creating theranostic NPs that integrate both therapeutic and diagnostic functions, as well as multifunctional NPs capable of targeting, imaging, and therapy to streamline treatment processes. Exploring the role of metal NPs in immunotherapy and their potential in combination therapies with existing cancer treatments can further enhance therapeutic outcomes. Advances in NP design and engineering, such as stability, biocompatibility, and targeting capabilities, will be crucial, as will the development of new fabrication and functionalization methods. Finally, strategies for clinical translation, such as designing clinical trials and establishing regulatory guidelines, are essential to ensure the safe and effective application of metal NPs in cancer treatment, thereby accelerating their integration into clinical practice.
